# Transfer of HTLV-1 p8 and Gag to target T-cells depends on VASP, a novel interaction partner of p8

**DOI:** 10.1371/journal.ppat.1008879

**Published:** 2020-09-30

**Authors:** Norbert Donhauser, Eileen Socher, Sebastian Millen, Stefanie Heym, Heinrich Sticht, Andrea K. Thoma-Kress

**Affiliations:** 1 Institute of Clinical and Molecular Virology, Friedrich-Alexander-Universität Erlangen-Nürnberg (FAU), Erlangen, Germany; 2 Division of Bioinformatics, Institute of Biochemistry, Friedrich-Alexander-Universität Erlangen-Nürnberg (FAU), Erlangen, Germany; Duke University Medical Center, UNITED STATES

## Abstract

The Human T-cell leukemia virus type 1 (HTLV-1) orf I-encoded accessory protein p8 is cleaved from its precursor p12, and both proteins contribute to viral persistence. p8 induces cellular protrusions, which are thought to facilitate transfer of p8 to target cells and virus transmission. Host factors interacting with p8 and mediating p8 transfer are unknown. Here, we report that vasodilator-stimulated phosphoprotein (VASP), which promotes actin filament elongation, is a novel interaction partner of p8 and important for p8 and HTLV-1 Gag cell-to-cell transfer. VASP contains an Ena/VASP homology 1 (EVH1) domain that targets the protein to focal adhesions. Bioinformatics identified a short stretch in p8 (amino acids (aa) 24–45) which may mediate interactions with the EVH1 domain of VASP. Co-immunoprecipitations confirmed interactions of VASP:p8 in 293T, Jurkat and HTLV-1-infected MT-2 cells. Co-precipitation of VASP:p8 could be significantly blocked by peptides mimicking aa 26–37 of p8. Mutational studies revealed that the EVH1-domain of VASP is necessary, but not sufficient for the interaction with p8. Further, deletion of the VASP G- and F-actin binding domains significantly diminished co-precipitation of p8. Imaging identified areas of partial co-localization of VASP with p8 at the plasma membrane and in protrusive structures, which was confirmed by proximity ligation assays. Co-culture experiments revealed that p8 is transferred between Jurkat T-cells via VASP-containing conduits. Imaging and flow cytometry revealed that repression of both endogenous and overexpressed VASP by RNA interference or by CRISPR/Cas9 reduced p8 transfer to the cell surface and to target Jurkat T-cells. Stable repression of VASP by RNA interference in chronically infected MT-2 cells impaired both p8 and HTLV-1 Gag transfer to target Jurkat T-cells, while virus release was unaffected. Thus, we identified VASP as a novel interaction partner of p8, which is important for transfer of HTLV-1 p8 and Gag to target T-cells.

## Author summary

The delta-retrovirus Human T-cell leukemia virus type 1 encodes the accessory protein p8, which is generated by proteolytic cleavage from p12. Earlier work has shown that p8 enhances the formation of cellular conduits between T-cells, is transferred through these conduits to target T-cells and increases HTLV-1 transmission. It was suggested that p8 dampens T-cell responses in target T-cells, thus facilitating HTLV-1 infection. Our work sheds light on the mechanism of p8 transfer to target T-cells. We show that vasodilator-stimulated phosphoprotein (VASP), a novel interaction partner of p8, contributes to transfer of p8 to target T-cells. Mechanistically, VASP is crucial for recruitment of p8 to the cell surface. Since VASP is known to promote elongation of actin filaments by preventing them from capping, interactions of p8 with VASP are an elegant strategy to exploit the host cell machinery for being transported to the cell surface, and as a consequence, to other cells. Given that VASP is also important for cell-to-cell transfer of the HTLV-1 Gag protein, our work proposes that VASP is a new cellular target to counteract HTLV-1 cell-to-cell transmission.

## Introduction

Human T-cell leukemia virus type 1 (HTLV-1), a delta-retrovirus infecting ca. 5–10 million people worldwide, is transmitted via cell-containing body fluids such as blood products, semen, and breast milk [1,2]. Upon binding to its receptor [3], HTLV-1 infects its target cells, CD4^+^ T-cells, and to a less extent CD8^+^ T-cells, dendritic cells (DC), or monocytes [4–6]. HTLV-1 integrates into the host cell genome and persists *in vivo* mainly in its provirus form (9.1 kb), which is flanked by long terminal repeats (LTR). In addition to structural proteins and enzymes common for retroviruses, HTLV-1 encodes regulatory (Tax, Rex) and accessory (p12/p8, p13, p30, HBZ) proteins [7]. HTLV-1 replicates either by infecting new cells or by mitotic division and clonal proliferation of infected CD4^+^ T-cells [8]. During their lifetime, approximately 1–5% of HTLV-1 infected individuals develop adult T-cell leukemia/lymphoma (ATL), and another 0.3–4% HTLV-1-associated myelopathy/tropical spastic paraparesis (HAM/TSP) [9].

Cell-free transmission of HTLV-1 is inefficient as free virions can hardly be detected in infected individuals and are poorly infectious for most cell types [2,4,10–13]. Efficient infection of CD4^+^ T-cells requires cell-cell contacts, and virus propagation from cell-to-cell depends on specific interactions between cellular and viral proteins [2]. Two types of cell-cell contacts seem to be critical for HTLV-1 transmission: (1) tight cell-cell contacts [14] and, (2) long-distance connections including cellular conduits [15] and tunneling nanotubes (TNTs) [16]. For transmission at tight cell-cell contacts, two non-exclusive mechanisms of virus transmission at the virological synapse (VS), a virus-induced specialized cell-cell-contact, have been proposed: polarized budding of HTLV-1 into synaptic clefts [14] and cell surface transfer of viral biofilms [17]. For formation of the VS and the viral biofilm, the viral transactivator Tax plays a major role [18,19]. For transmission via cellular conduits, however, the accessory protein p8 is a prerequisite [15,16].

The HTLV-1 p8 protein is a cleavage product of the viral accessory p12 protein encoded by open reading frame (orf) I [20]. The precursor protein p12 localizes to the endoplasmatic reticulum (ER) and to the Golgi apparatus [21,22]. p12 is post-translationally modified by a two-step proteolytic cleavage: the first cleavage between amino acid (aa) 9/10 removes an ER-retention signal, which allows trafficking of the protein to the Golgi. The second cleavage occurs between aa 29/30 resulting in the p8 protein [20]. p8 is a 70 aa protein that localizes to the cytoplasm and is recruited to lipid rafts and the immunological synapse upon T-cell receptor ligation [20,23]. Upon expression in T-cells, p8 increases the number of T-cell contacts through enhancement of lymphocyte function-associated antigen-1 (LFA-1) clustering on the surface of T-cells [15]. Additionally, p8 increases both the number and length of cellular conduits between T-cells. These conduits are membrane extensions that are supposed to be formed by directed outgrowth of a filopodium-like protrusion or TNT toward a neighboring cell [15,16]. p8 enhances viral infectivity, and thus, supports viral persistence *in vivo* [24], a function that had been earlier attributed to its precursor p12 [25–27]. Mechanistically, the increase in virus transmission is thought to be mediated by the ability of p8 to induce intercellular conduits [15]. Since viral particles are detectable at the contact site between conduits and target cells, but no “surfing” of HTLV-1 virions on cellular conduits has been observed [15], it is assumed that HTLV-1 buds from the conduit towards the target cell at a “mini-VS” [28]. Interestingly, p8 is also transferred to neighboring cells within minutes, invades target cells [15,29], and is supposed to induce T-cell anergy by decreasing T-cell receptor signaling [23]. The latter had been experimentally shown with constructs expressing both p12 and p8 [23]. Together, these potential functions of p8 could favor persistence of HTLV-1 in an immune competent host [15,30]. While *in vitro* studies using proviral clones lacking orf I suggested that p12 is not essential for viral replication [31], *in vivo* studies in macaques support the notion that p8 and p12 are important for viral persistence and spread. Moreover, productive infection of monocytes depends on the expression of p8 and p12 proteins [24,32]. Since it is unknown how p8 is transferred to other cells, we sought for identification of cellular interaction partners of p8 that could mediate p8-transfer, and potentially, HTLV-1 transmission.

Vasodilator-stimulated phosphoprotein (VASP) is a member of the Ena/VASP homology 1 (EVH1) family and was originally identified as a substrate for cAMP- and cGMP-dependent protein kinases in human platelets [33–35]. Many EVH1-domain containing proteins are closely associated with actin-based structures and actin remodeling [36]. VASP is composed of an N-terminal EVH1 domain that mediates interaction with proline-rich motifs in interacting proteins that help to position the Ena/VASP tetramer near the plasma membrane. The central part of VASP is proline-rich and harbors binding sites for profilin which, in turn, binds G-actin monomers and is thought to supply actin monomers at the barbed ends of actin filaments [37,38]. The EVH2 domain contains a G-actin-binding site (GAB) that mediates binding of globular actin, and an F-actin-binding site (FAB) mediating interaction with filamentous actin. At the very C-Terminus, a coiled-coil domain gives rise to tetramerization [38]. Thus, VASP prevents elongating actin-filaments from capping thereby promoting filament elongation [38]. VASP is strongly enriched in focal contacts and along stress fibers; beyond, it modulates the morphology of membrane protrusions such as filopodia and lamellipodia, and thus, influences cell motility [34,38]. VASP is also involved in actin-based motility of pathogens like the bacteria *Listeria* and *Shigella* and of Vaccinia virus [39–41].

Here we show that VASP is a novel interaction partner of HTLV-1 p8 and important for p8 and HTLV-1 Gag transfer between cells. Mapping the VASP:p8 interaction revealed that at least three different domains in VASP and a short sequence stretch in p8 (aa 26–37) are important for the VASP:p8 interaction. Functionally, repression of VASP by gene editing strategies demonstrated that VASP is not only crucial for the transfer of p8 to target T-cells, but also of HTLV-1 Gag, suggesting that HTLV-1 cell-to-cell transmission depends on VASP. Taken together, interactions of p8 with VASP could contribute to viral spread and the establishment of persistent HTLV-1 infections.

## Results

### Identification of VASP as a novel interaction partner of p8 based on bioinformatics predictions

**Fig 1:**
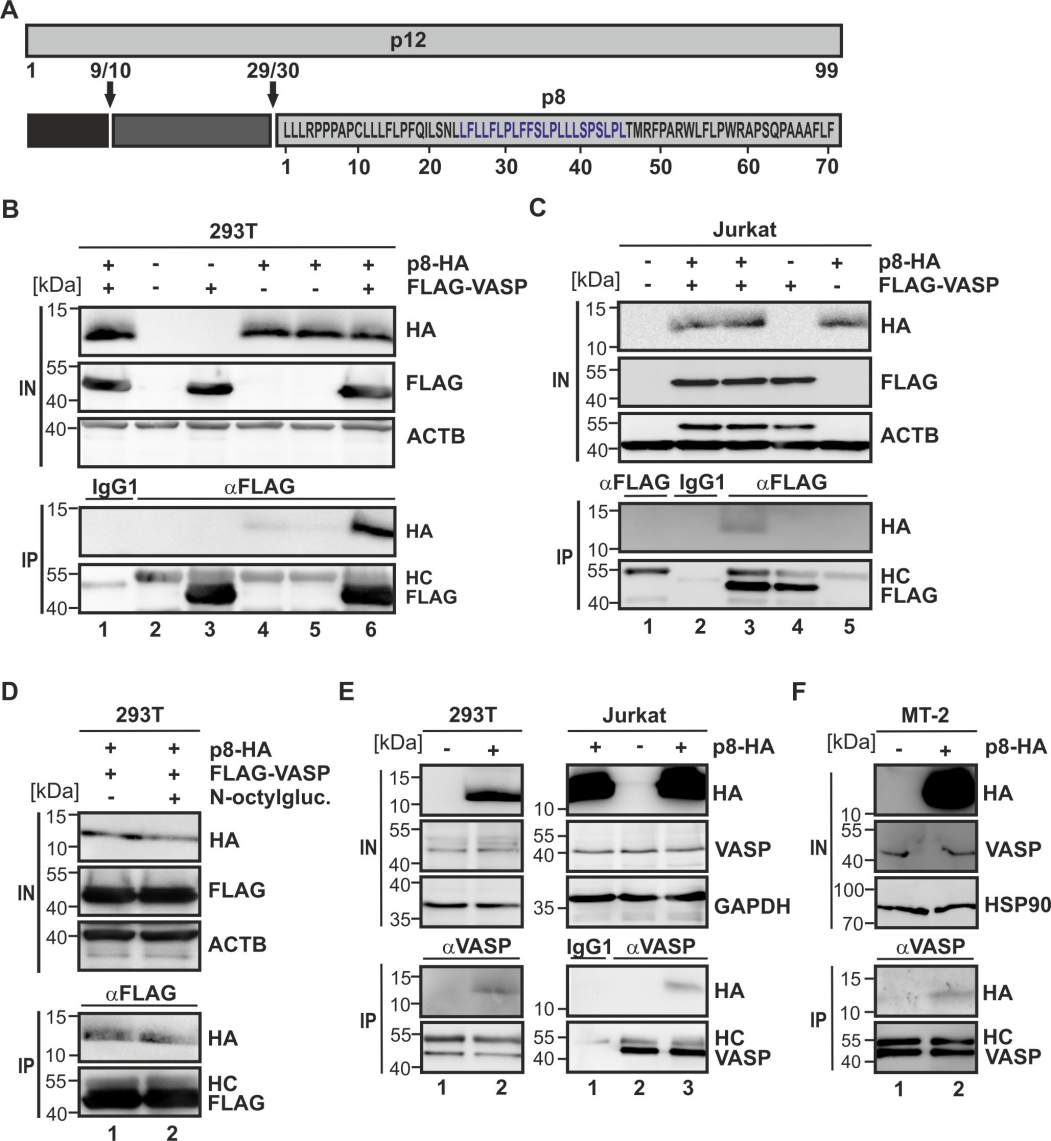
VASP is a novel interaction partner of p8. **(A)** Scheme of the HTLV-1 p8 protein and its precursor p12. A motif mediating potential interactions with the VASP EVH1 domain is highlighted in blue. **(B)** 293T cells or **(C)** Jurkat T-cells were transfected with expression plasmids p8-HA and FLAG-VASP or empty control vectors. After 48 h, cells were lysed and 10% of the lysates were taken as input (IN). Co-immunoprecipitations (IPs) were performed using anti-FLAG antibodies or isotype-matched control antibodies (IgG). Immunoblots are shown. HC, heavy chain. **(D)** 293T cells were transfected with expression plasmids p8-HA and FLAG-VASP. After 48 h, cells were lysed and 10% of the lysates were taken as input. Lysates were treated without (lane 1) or with 60 mM N-octyl-β-D-glucoside (N-ocytlgluc.; lane 2). IPs were performed using anti-FLAG antibodies. Immunoblots are shown. **(E)** 293T, Jurkat or **(F)** HTLV-1-infected MT-2 cells were transfected with expression plasmids p8-HA. After 48 h, cells were lysed and 10% of the lysates were taken as input. Endogenous VASP was precipitated using anti-VASP antibodies. Immunoblots are shown.

To predict putative interaction partners of p8, the amino acid sequence of p8 (Fig 1A) was analyzed, revealing that p8 is a highly hydrophobic protein that is also rich in prolines (proline content 18.6%). This prompted us to search for proline-rich motifs (PRMs) in the p8 sequence, because such short linear motifs can mediate protein-protein interactions with larger globular domains. At least five different families of adaptor domains are known that recognize peptides containing PRMs: Src-homology 3 (SH3) domains, WW domains, Ena/VASP homology 1 (EVH1) domains, GYF domains, and Ubiquitin E2 variant (UEV) domains [42]. The EVH1 family seemed to be the most promising candidate as putative interaction partner of p8, because many EVH1-containing proteins are closely associated with actin-based structures and actin remodeling [36,43]. Especially, vasodilator-stimulated phosphoprotein (VASP) and some members of the Wiskott-Aldrich Syndrome Protein (WASP) family as representatives of the EVH1-domain-containing proteins seemed to be interesting candidates because it is thought that VASP promotes actin filament elongation by preventing elongating actin-filaments from capping and by recruiting profilin-actin complexes [38]. Therefore, a putative VASP:p8 interaction could offer an explanation for the p8-mediated actin-dependent cell-cell conjugation, for protrusion formation in HTLV-infected cells, and for the transport of p8 to other cells [15,16,29]. In order to investigate whether p8 has potential PRMs which could bind to the VASP EVH1 domain, we defined a binding pattern based on the results of an experimental peptide screen published earlier (*see* Materials and Methods) [43]. A pattern search against the p8 sequence results in a large number of hits, which are mainly detected for the sequence stretch _24_LFLLFLPLFFSLPLLLSPSLPL_45_ (Fig 1A, highlighted in blue). This observation supports the idea that p8 interacts with the VASP EVH1 domain and that this interaction is most likely mediated by residues 24–45. To verify the predictions, HA-tagged p8 expression constructs were co-expressed with FLAG-tagged VASP expression constructs in 293T cells (Fig 1B). Compared to the controls (lanes 2–5), precipitation of FLAG-VASP using FLAG-specific antibodies resulted in co-precipitation of p8-HA (lane 6), while an isotype-matched control antibody did not co-precipitate p8-HA (lane 1). These findings were confirmed in Jurkat T-cells (Fig 1C), where precipitation of FLAG-VASP resulted in co-precipitation of p8 (Fig 1C, lane 3), too. Since p8 localizes at the plasma membrane [20], co-IPs were also performed in presence of N-octyl-β-D-glucoside, which is known to solubilize membrane proteins and to enhance selectivity of immunoprecipitations [44]. Irrespective of the absence (Fig 1D, lane 1) or presence (Fig 1D, lane 2) of N-octyl-β-D-glucoside, p8 co-precipitated with VASP in 293T cells, suggesting that the VASP:p8 interaction is specific. Further, precipitation of endogenous VASP protein using VASP-specific antibodies upon overexpression of p8-HA expression constructs in 293T-cells and Jurkat T-cells also resulted in co-precipitation of p8-HA (Fig 1E, left blot, lane 2; and right blot, lane 3). Finally, we analyzed the chronically HTLV-1-infected T-cell line MT-2, which, based on sequence, should predominantly express p8 [16], however, at levels below the limit of detection by western blot [45,46]. To circumvent these limitations and since specific antibodies targeting endogenously expressed p8 only have not been described yet, we decided to overexpress p8-HA in MT-2 cells. Confirming our earlier observations, we could detect co-precipitation of endogenous VASP with p8-HA also in MT-2 cells (Fig 1F, lane 2). In summary, these data confirm the bioinformatics predictions and show that VASP is indeed a novel interaction partner of p8 in different cell lines.

### The EVH1-domain and the G- and F-actin-binding domains of VASP are required for the VASP:p8 interaction

**Fig 2:**
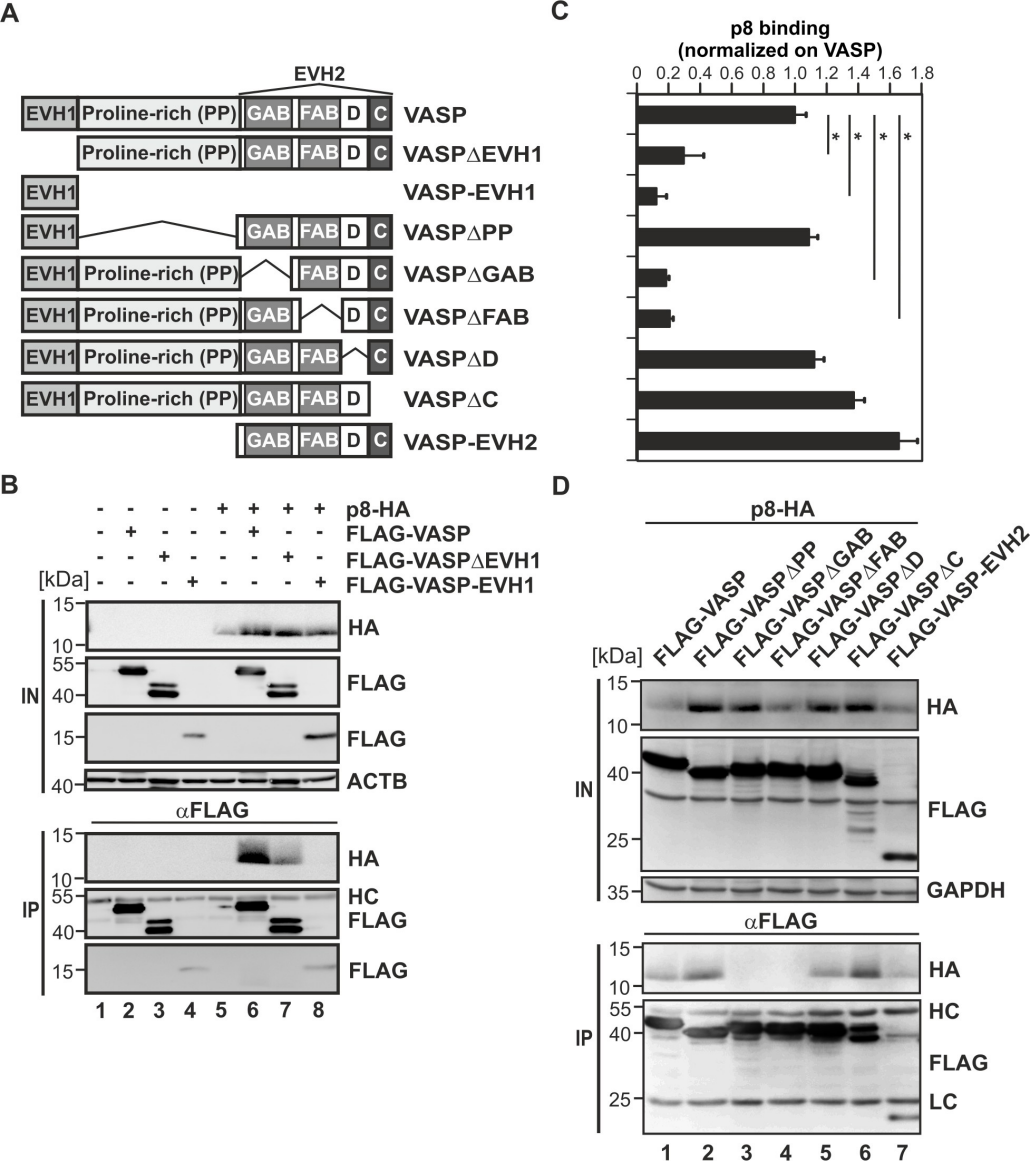
The EVH1-domain and the F- and G-actin binding domains of VASP are required for the interaction with p8. **(A)** Domain structure of VASP protein and mutants. **(B)** 293T cells were transfected with expression plasmids p8-HA and FLAG-VASP, FLAG-VASPΔEVH1, FLAG-VASP-EVH1 or the respective empty control vectors. After 48 h, cells were lysed and 10% of the lysates were taken as input (IN). Co-immunoprecipitations (IPs) were performed using anti-FLAG antibodies. Immunoblots are shown. HC, heavy chain. One representative out of four independent experiments is shown. **(C)** Densitometry was performed to quantitate the amount of p8-HA after precipitation and values were normalized on the respective FLAG-VASP expression in the input. p8 binding to VASP wildtype was set to 100%. Bars indicate the means of four independent experiments ± SE and values were compared to VASP wildtype using Student’s t-test. * indicates p<0.05. **(D)** 293T cells were transfected with expression plasmids p8-HA and FLAG-VASP, FLAG-VASPΔPP, FLAG-VASPΔGAB, FLAG-VASPΔFAB, FLAG-VASPΔD, FLAG-VASPΔC or FLAG-EVH2. IPs were performed as described in **(A)**. One representative out of four independent experiments is shown.

VASP contains an N-terminal EVH1 domain which mediates interaction with proline-rich motifs in interacting proteins that help to position the Ena/VASP tetramer near the plasma membrane (Fig 2A). The central part of VASP is proline-rich and harbors binding sites for profilin which, in turn, binds globular actin monomers and is thought to supply actin monomers at the barbed ends of actin filaments. The EVH2 domain contains a G-actin-binding site (GAB) that mediates binding of globular actin, and an F-actin-binding site (FAB) leading to interaction with filamentous actin. At the very C-Terminus, a coiled-coil domain is located that is important for tetramerization (Fig 2A) [38]. Since bioinformatics predicted an interaction between p8 and the EVH1-domain of VASP, we analyzed the relevance of the EVH1 domain for the interaction between VASP and p8 in more detail. Expression plasmids of p8-HA were co-expressed with the respective FLAG-tagged VASP expression constructs (VASP wildtype, VASPΔEVH1, or VASP-EVH1) in 293T cells and co-immunoprecipitations were performed. Analysis of the input fraction revealed that all constructs were expressed as expected (Fig 2B). Expression of FLAG-VASP (Fig 2B, lanes 2, 6; input) and—visible more pronouncedly—of FLAG-VASPΔEVH1 (Fig 2B, lanes 3, 7; input), showed a second, slower migrating band, which may result from VASP phosphorylation [47], and which was not visible when expressing the VASP-EVH1 domain alone (Fig 2B, lanes 4, 8; input). Precipitation of the FLAG-tagged VASP constructs revealed that only wildtype VASP resulted in a strong co-precipitation of p8 (Fig 2B, lane 6). In contrast, a VASP mutant lacking the EVH1 domain (VASPΔEVH1) was severely impaired in co-precipitating p8 (Fig 2B, lane 7) confirming the bioinformatics predictions. However, to our surprise, precipitation of the EVH1 domain alone (VASP-EVH1) was not sufficient to co-precipitate p8 efficiently (Fig 2B, lane 8). Densitometry confirmed these findings and showed that both VASPΔEVH1 and VASP-EVH1 were significantly impaired in co-precipitating p8 compared to VASP wildtype (Fig 2C; p<0.05; n = 4). Taken together, the EVH1 domain of VASP is necessary, but not sufficient to interact with p8 suggesting that the EVH1 domain may mainly be important for the proper localization of VASP in close proximity to p8 at the cell periphery in living cells [38], for recruiting of p8, or for resolving an unfavorable protein conformation. Thus, other domains of VASP might contribute to the interaction with p8.

To further map the interface of the VASP:p8 interaction, we analyzed additional VASP-mutants (Fig 2A), which were devoid of the proline-rich region (VASPΔPP), which carried deletions in the EVH2 domain (VASPΔGAB, VASPΔFAB, VASPΔD, VASPΔC), or which expressed the EVH2-domain only (VASP-EVH2). Upon co-expression of p8-HA and the respective FLAG-tagged VASP-mutants in 293T-cells, co-immunoprecipitations using FLAG-specific antibodies followed by western blots were performed (Fig 2D) and densitometry was applied (Fig 2C). These experiments confirmed that p8 co-precipitates with VASP wildtype (Fig 2D, lane 1). Interestingly, co-immunoprecipitations revealed that two additional domains, GAB and FAB, are crucial for the VASP:p8 interaction (Fig 2D, lanes 3–4) since deletion of the G- and F-actin binding region in VASP severely impaired co-precipitation of p8 (Fig 2C, p<0.05; n = 4). Conveniently, a VASP-mutant expressing the EVH2 domain (VASP-EVH2), which contains GAB and FAB, was not impaired in binding p8 compared to VASP wildtype (Fig 2D, lanes 7 and 1; Fig 2C) taking into account the lower expression levels of VASP-EVH2 in the input fraction. This suggests that actin-binding is required for the VASP:p8 interaction. Further, mutations of the proline-rich region (VASPΔPP), the coiled-coil domain (VASPΔC), or of a region adjacent to the coiled-coil domain (VASPΔD) did not reduce binding of p8 (Fig 2D, lanes 2, 5, 6; Fig 2C). Taken together, our findings show that not only the EVH1 domain, but also the G-and the F-actin binding domains of VASP contribute to the interaction with p8. Further, the EVH2 domain seems to dominate over the EVH1 domain in interacting with p8 since a mutant expressing the EVH2 domain alone is not impaired in precipitating p8.

### Peptides mimicking (aa) 26–37 of p8 block the VASP:p8 interaction

**Fig 3:**
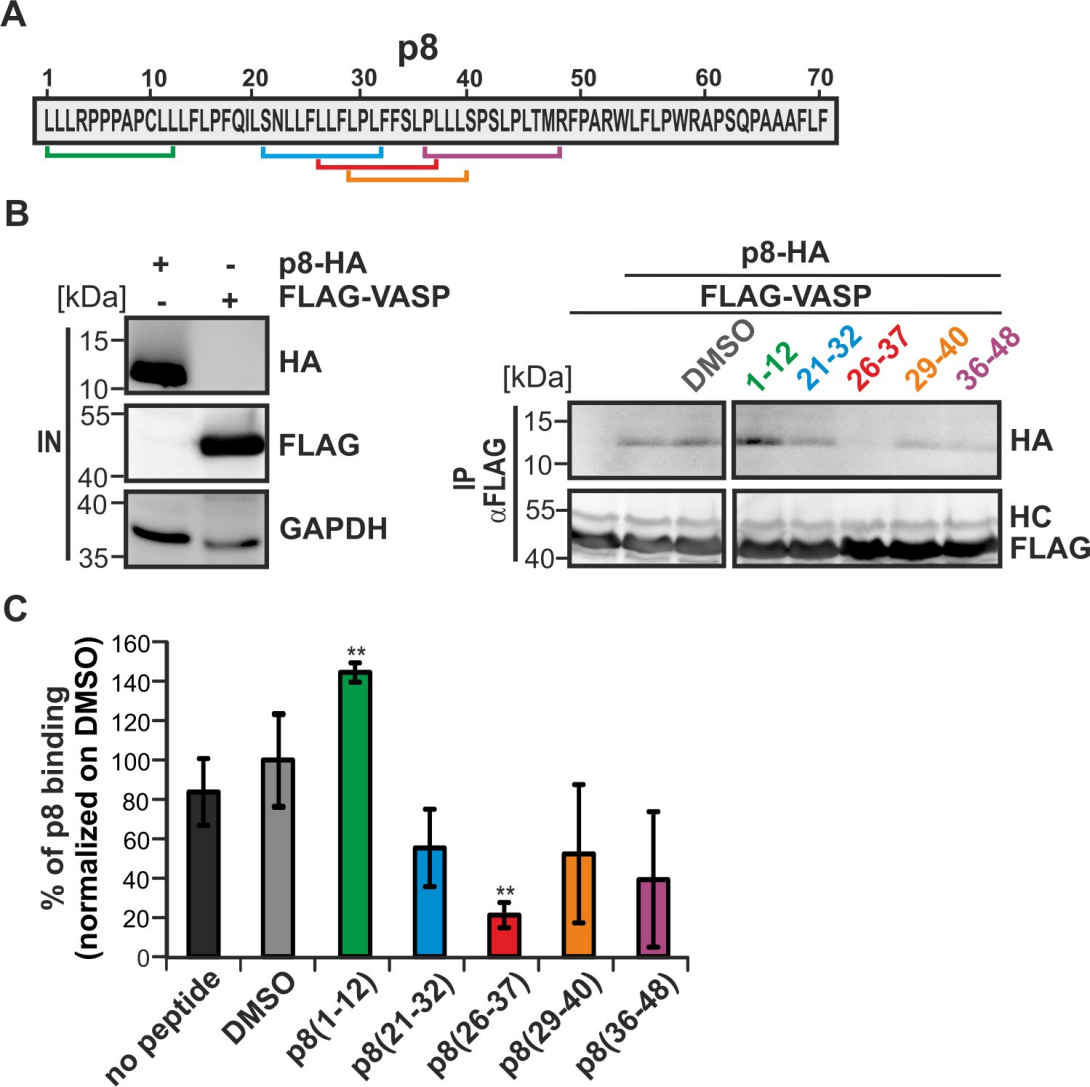
p8-mimicking peptides reduce the interaction between p8 and VASP. **(A)** Amino acid sequence of p8. Bars indicate location of competitive p8 inhibitory peptides. **(B)** p8-HA and FLAG-VASP were expressed individually in 293T cells. After 48 h, cells were lysed and 10% of the cell lysate were taken as input (IN). The remaining lysates were co-incubated with the peptides shown in **(A)**, or with the solvent control DMSO for 1.5 h at 20°C. Thereafter, lysates were mixed for 1.5 h, and co-precipitations (anti-FLAG) were performed (IP). Representative immunoblots are shown. The blot (IP) was cut due to technical reasons. HC, heavy chain. **(C)** The impact of p8-mimicking peptides on binding of p8-HA after precipitation of FLAG-VASP was assessed by densitometry. DMSO was set as 100%. The means of four independent experiments ± SE were compared to DMSO-treated cells using Student’s t-test. ** indicates p<0.01.

Having identified critical regions in VASP that are required for the interaction with p8, we next analyzed p8 in more detail. In order to evaluate whether the proline-rich sequence stretch _24_LFLLFLPLFFSLPLLLSPSLPL_45_ of p8, which led to the prediction of VASP as a novel interaction partner of p8, is important for the co-precipitation of VASP:p8, competitive inhibitory peptides were designed (*see* Materials and Methods). These short peptides cover the predicted EVH1-binding motif regions of p8 and overlap each other to allow a finer mapping of the interacting region (Fig 3A). The peptide p8(1–12) was designed as a negative control, because it covers also a proline-rich sequence stretch in the N-Terminus of p8, however, this region was not predicted by the pattern search as an EVH1-binding motif. To test whether the interaction between VASP and p8 is affected by these peptides, 293T cells were transfected with expression constructs of either p8-HA or FLAG-VASP. After lysis, 10% of the lysate was taken as input control and western blot confirmed expression of p8-HA and FLAG-VASP (Fig 3B, left part). The rest of the cell lysates was incubated with the respective peptides, which were used at comparable dilutions that had been determined before. After 1.5 h of co-incubation with the peptides, the p8-and VASP-lysates were co-incubated for another 1.5 h, and thereafter, precipitations (anti-FLAG) were performed. Analysis of the co-immunoprecipitations by western blot (Fig 3B, right part) and subsequent densitometry (Fig 3C) revealed that co-precipitation of p8 could be blocked by all peptides overlapping with the aforementioned sequence stretch _24_LFLLFLPLFFSLPLLLSPSLPL_45_ of p8, while the control peptide p8(1–12) did not block but rather enhanced the VASP:p8 interaction. Among the peptides tested, peptide p8(26–37) led to a significant reduction (Fig 3C; p<0.01; n = 4) of co-precipitated p8. These *in vitro* data suggest that the peptide p8(26–37) may recognize the EVH1 domain of VASP, and thus, may also interfere with the VASP:p8 interaction *in vivo*. Taken together, fine-mapping of the VASP:p8 interaction identified critical regions in VASP and p8 which seem to be important for the interaction.

### p8 and VASP partially co-localize at the plasma membrane and in protrusive structures between cells

**Fig 4:**
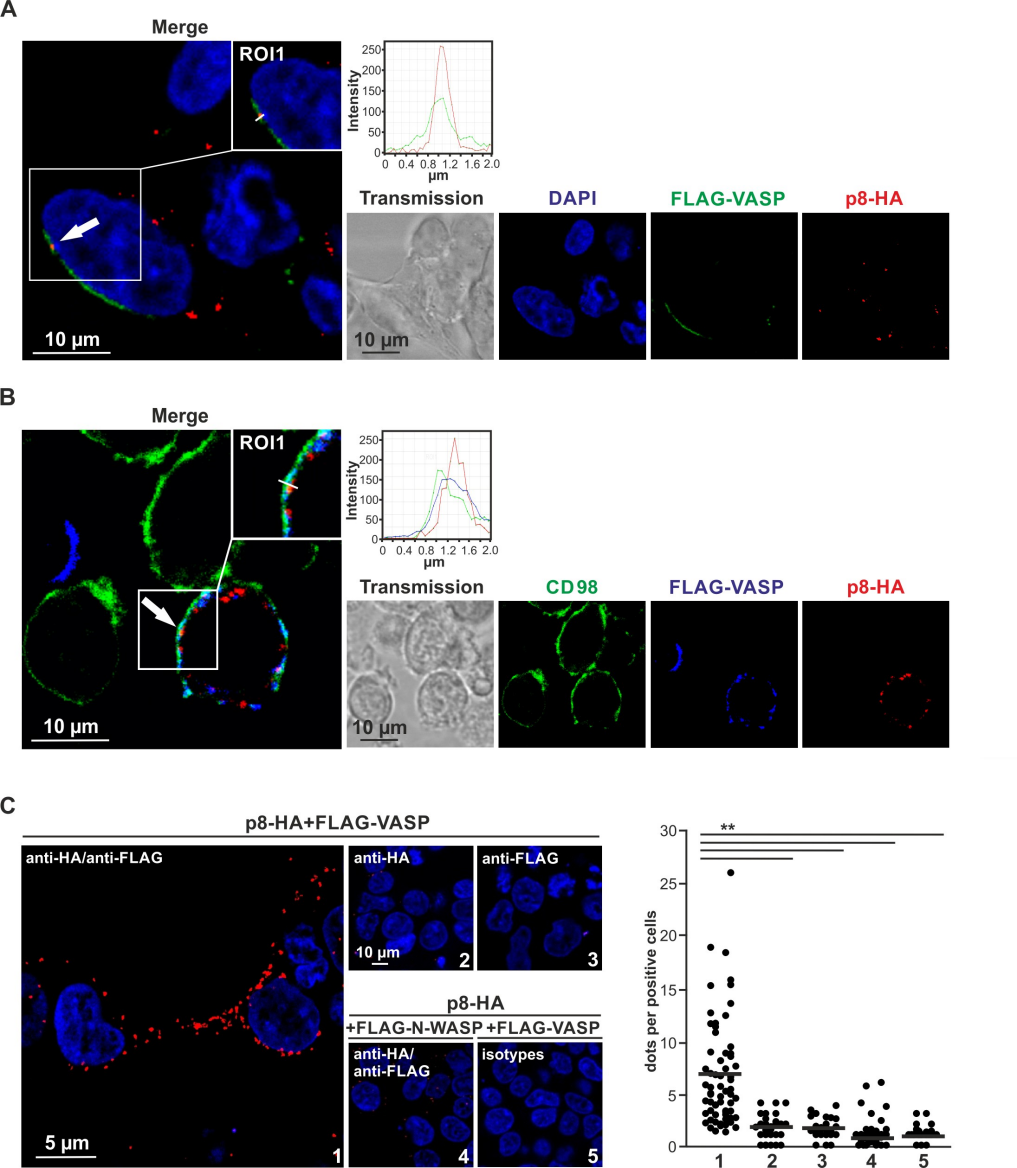
p8 and VASP partially co-localize at the plasma membrane. Confocal laser scanning microscopy of **(A)** 293T cells (seeded on coverslips), and **(B)** Jurkat T-cells after co-transfection of p8-HA and FLAG-VASP expression plasmids. **(A)** Stains of FLAG-VASP (green), p8-HA (red), the cell nucleus (DAPI, blue), the merge of all three stains and transmitted light are shown. Regions of interest (ROI) are shown and highlighted in insets. Solid arrows indicate co-localizations of p8-HA and FLAG-VASP. Graphs show the fluorescence intensities of FLAG-VASP- and p8-HA-specific fluorescence along the ROI. **(B)** Stains of the plasma membrane marker CD98 (green), FLAG-VASP (blue), p8-HA (red), the merge of all stains and transmitted light are shown. ROIs are shown and highlighted in insets. Solid arrows indicate co-localizations of p8-HA, FLAG-VASP, and CD98. Graphs show the fluorescence intensities of FLAG-VASP-, p8-HA-, and CD98-specific fluorescence along the ROI. **(C)** Proximity ligation assay (PLA) in 293T cells after co-expression of p8-HA and FLAG-VASP or the control FLAG-N-WASP. PLA was performed following incubation with primary antibodies targeting either HA, FLAG, both FLAG and HA, or the respective isotype controls. Red dots indicate co-localization. Left side: representative stains. Right side: Dots per positive cell were counted in four to ten optical fields per experiment analyzing 28–64 different cells in three independent experiments. Mean values are indicated by a line. Values were compared using Student’s t-test. **, p<0.01.

**Fig 5:**
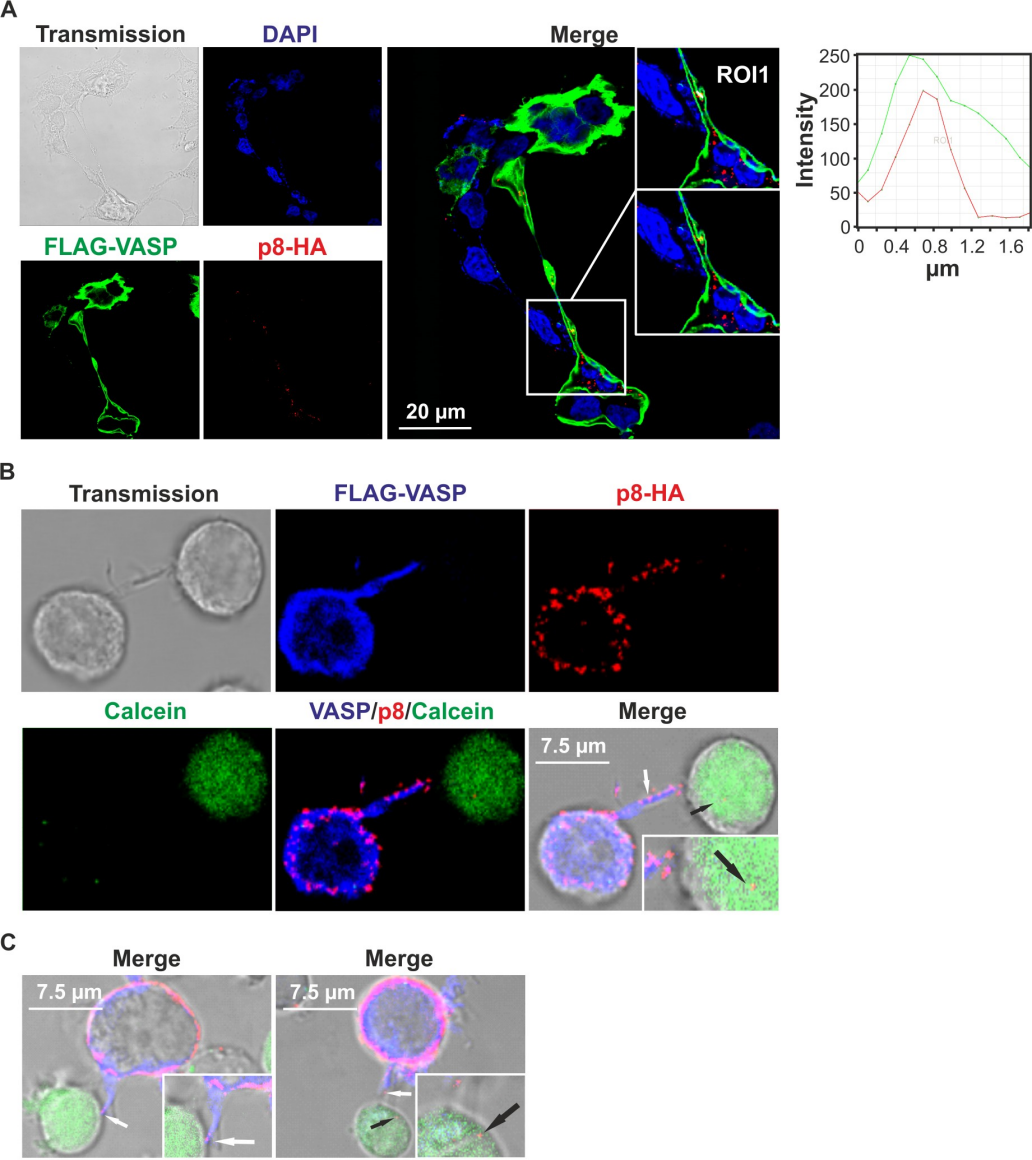
p8 and VASP partially co-localize in protrusive structures between cells and p8 is transferred to target T-cells via VASP-containing protrusions. **(A)** Confocal laser scanning microscopy of 293T cells upon co-transfection of p8-HA and FLAG-VASP expression plasmids. Stains of FLAG-VASP (green), p8-HA (red), the cell nuclei (DAPI, blue), the merge of all three stains and transmitted light are shown. Regions of interest (ROI) within a protrusive structure are shown and graphs show the fluorescence intensities of FLAG-VASP- and p8-HA-specific fluorescence along the ROI. **(B)** Jurkat T-cells were co-transfected with expression plasmids p8-HA, FLAG-VASP and pMACS-LNGFR. After 48 h, transfected cells were enriched by magnetic separation using LNGFR-specific microbeads and co-cultured with untransfected Jurkat T-cells pre-stained with the live cell marker Calcein (green) on poly-L-lysine-coated coverslips for 30 min at 37°C. Immunofluorescence stainings of FLAG-VASP (blue), p8-HA (red), the merge of all stainings and transmitted light are depicted. White arrow: p8 co-localizing with VASP in a protrusion; black arrow: p8 in co-cultured target Jurkat T-cell. **(C)** Two additional examples of merges showing p8 co-localizing with VASP in a protrusion (white arrows) and p8 in co-cultured target Jurkat T-cells (black arrows) as described in **(B)**.

To get insights into the subcellular localization of p8 and VASP, 293T cells were transiently transfected with FLAG-VASP and p8-HA expression plasmids and subjected to immunofluorescence stains followed by confocal laser scanning microscopy (Fig 4A). Analysis of the images revealed that VASP is highly expressed and exclusively located in the cytoplasm and accumulated at the peripheral cytoplasm confirming earlier observations in different cell types [48]. Contrary, p8 is distributed in dot-like structures in the cytoplasm and at the plasma membrane which is in line with earlier findings [20]. However, we also found areas of partial co-localization of p8 with VASP at the plasma membrane in dot-like structures (Fig 4A, solid white arrow) supporting the data obtained by co-immunoprecipitation (Fig 1B). Analysis of the p8-HA- and FLAG-VASP-specific fluorescence intensities revealed a parallel distribution of the respective fluorescences along a region of interest (ROI) supporting the notion that both proteins co-localize and interact (Fig 4A, diagram). Next, we analyzed a more physiological cell type with respect to HTLV-1 infections and overexpressed FLAG-VASP and p8-HA in the CD4^+^ T-cell line Jurkat (Fig 4B). Further, we stained the plasma membrane marker CD98. Confocal laser scanning microscopy showed co-localization of VASP with CD98 and confirmed the partial co-localization of VASP and p8 at the plasma membrane also in Jurkat T-cells (Fig 4B, solid white arrow), which was again supported by a parallel distribution of FLAG-VASP-, p8-HA-, and CD98-specific fluorescence intensities along a ROI at the plasma membrane (Fig 4B, diagram). Interestingly, we also observed protrusive structures between cells, which contained both VASP and p8 (S1 Fig, open white arrow). To confirm the observed partial co-localizations between p8 and VASP on a quantitative basis, we performed proximity ligation assays (PLA), which detect protein-protein interactions closer than 40 nm *in situ* [49]. Briefly, p8-HA and FLAG-VASP were co-expressed in 293T cells. The cells were fixed, incubated with the indicated antibodies followed by incubation with PLA probes, ligation, and amplification. Interaction events between p8 and VASP could be detected as fluorescent spots. On average, we detected 7 spots per cell in p8- and VASP-expressing cells (Fig 4C, 1), while we only observed 1–2 spots in the controls including single stains (Fig 4C, 2;3) or isotype controls (Fig 4C, 5). Interestingly, the p8:VASP PLA signals also significantly exceeded weak signals upon co-expression of p8 with the membrane-located neuronal Wiskott-Aldrich syndrome protein (N-WASP), which is a VASP-related protein containing an EVH1-domain that does not interact with p8 (Fig 4C, 4).

Since earlier work found that p8 induces the number and length of cellular protrusions between cells [15,16] and we obtained initial hints that p8 and VASP may co-localize in protrusive structures (Fig 4B), we next had a closer look at the localization of VASP and p8 in protrusive structures in cell agglomerations. First, we analyzed 293T cells upon overexpression of FLAG-VASP and p8-HA by confocal laser scanning microscopy. We specifically searched for protrusive structures and indeed found p8-HA in dot-like structures throughout the protrusion (Fig 5A). Co-staining of FLAG-VASP revealed that VASP strongly accumulated along the protrusive structure and inspection of a ROI confirmed partial co-localization of p8 and VASP in the protrusion (Fig 5A, diagram). To analyze whether VASP- and p8-containing protrusions are also formed between T-cells, and whether p8 is transferred via these protrusions to other cells, we transfected Jurkat T-cells with p8-HA and FLAG-VASP expression plasmids. Additionally, we co-expressed a truncated version of LNGFR (low-affinity nerve growth factor receptor), which allowed us to enrich the number of transfected and p8-expressing cells within the population of transfected cells by magnetic separation [50]. After two days, transfected and enriched Jurkat T-cells (p8 donor cells) were co-cultured on poly-L-lysine-coated coverslips with untransfected Jurkat T-cells (target cells) that had been pre-stained with the live cell marker Calcein (green). Immunofluorescence of VASP (blue) and p8 (red) revealed three interesting findings (Fig 5B and 5C; S2 Fig): (1) p8 is distributed in dot like structures in the donor cells, especially near the plasma membrane, confirming earlier findings [20]. (2) p8 partially co-localizes with VASP in the transfected Jurkat T-cells and in VASP-containing protrusions that are found between Jurkat T-cells (white arrow). (3) p8 is transferred to target T-cells (black arrow), confirming earlier work [15,29], but this transfer presumably occurs via the VASP-containing-protrusions. Quantitative analysis revealed expression of either FLAG-VASP in 30.51% or of p8-HA in 5.08% of all protrusions, while 52.54% of all protrusions showed expression of both p8-HA and FLAG-VASP. Interestingly, p8-HA and FLAG-VASP co-localized in 83.87% of the p8-HA- and FLAG-VASP-double positive protrusions. Thus, these observations confirmed on the one hand that p8 and VASP interact, on the other hand, they prompted us to analyze whether VASP is important for p8-transfer to target T-cells.

### VASP is important for p8-transfer to target T-cells

**Fig 6:**
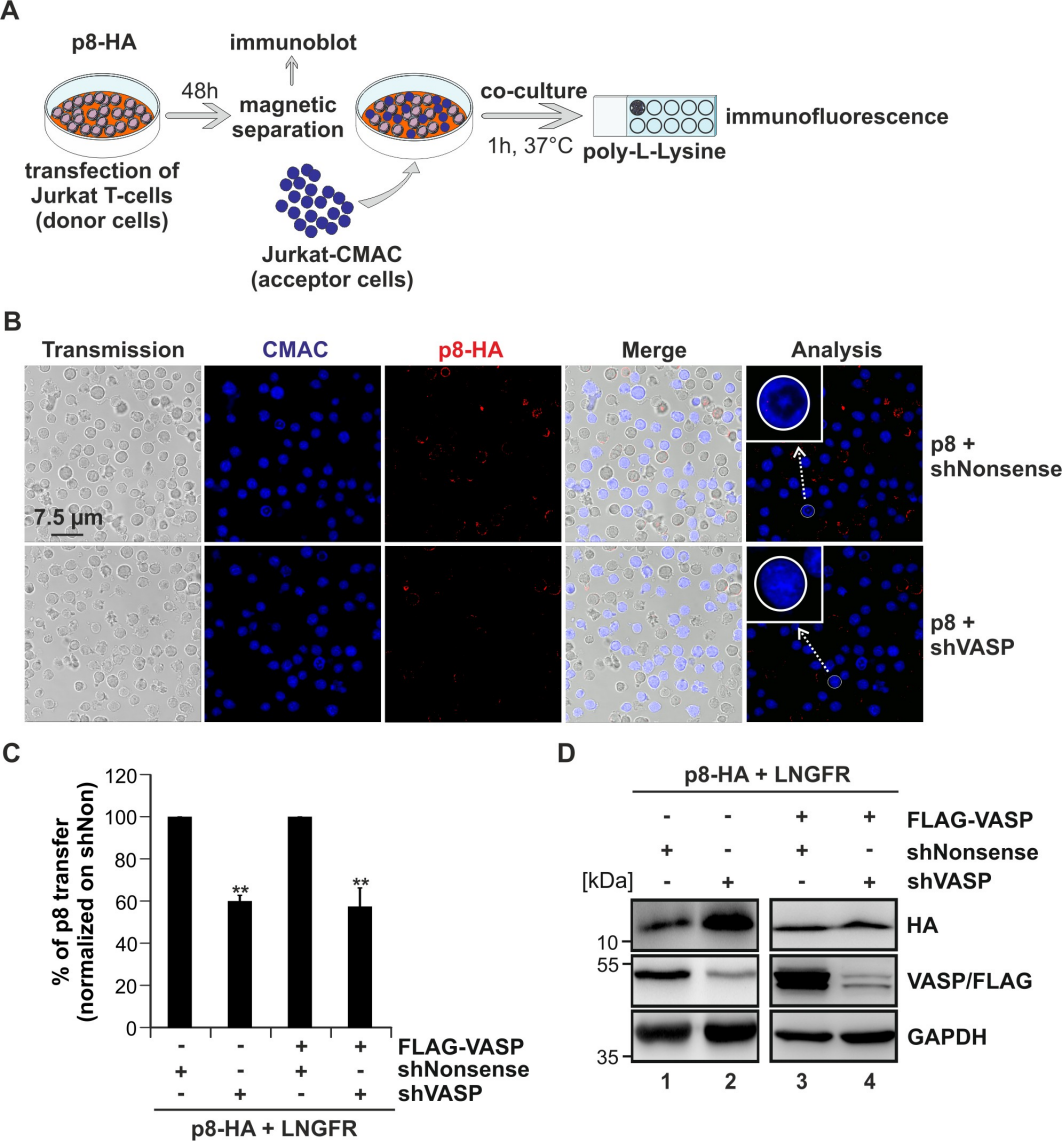
Repression of endogenous and overexpressed VASP reduces transfer of p8 to target T-cells. **(A)** Scheme of experimental setup. **(B-C)** Jurkat T-cells were transfected with expression plasmids p8-HA and pMACS-LNGFR. Additionally, FLAG-VASP or pEF (mock), shRNAs targeting VASP (shVASP1), or a control shRNA (shNonsense) were co-transfected. After 48 h, transfected cells were enriched by magnetic separation using anti-LNGFR-specific microbeads. Purified Jurkat T-cells were co-cultivated with acceptor Jurkat T-cells pre-stained with *CellTracker Blue CMAC Dye* (CMAC) on poly-L-lysine coated glass slides for 1 h at 37°C. Thereafter, cells were stained with HA- and FLAG-specific antibodies and the respective secondary antibodies. Slides were covered with *ProLong Gold antifade reagent* and analyzed by confocal microscopy. The numbers of cells expressing p8 (red) within the acceptor Jurkat T-cells (blue) were counted (see white circle in blow up as example). **(C)** The mean of five independent experiments (15–20 optical fields each) normalized on the number of p8-donor cells, and on the respective shNonsense ± SE is shown and was compared using Student’s t-test (**, p<0.01). **(D)** Repression of VASP was controlled by western blot analysis.

To elucidate whether the VASP:p8 interaction contributes to transfer of p8 to target cells, we designed and cloned shRNAs targeting VASP into the retroviral vector pSIREN-RetroQ-IRES-EGFP, which we already used previously to repress cellular gene expression [51,52]. Jurkat T-cells were transfected with p8-HA and shRNAs targeting VASP or a control shRNA (shNonsense). To discriminate between endogenous and overexpressed VASP, FLAG-VASP or the respective control vector pEF was co-transfected. Additionally, we co-expressed a truncated version of LNGFR to enrich the number of transfected and p8-expressing cells by magnetic separation (Fig 6A) [50]. After 48 h, transfected cells were enriched with LNGFR-specific microbeads and inspected by flow cytometry. Cells were either subjected to co-culture experiments with acceptor Jurkat T-cells that had been pre-stained with Cell Tracker Blue CMAC Dye (CMAC; Fig 6B and 6C) or to protein lysis and western blot analysis (Fig 6D). Upon co-culture with pre-stained Jurkat T-cells (Fig 6B, blue), all cells were stained for p8 using HA-specific antibodies (red), and the number of p8-donor cells (red fluorescence) and p8-acceptor cells (blue and red fluorescence; Fig 6B, white circles; S3 Fig, white circles) was determined. In summary, we analyzed approximately 6022 p8-expressing donor cells (ca. 1506 per experimental condition) manually, and we observed p8 in ca. 8.60% of all pre-stained target Jurkat T-cells after 1 h of co-culture. Thus, p8 is rapidly transferred to co-cultured target T-cells confirming earlier observations from our group and others [15,29]. Next, we quantitatively evaluated the impact of VASP on p8 transfer. As shown in representative examples depicted in Fig 6B and S3 Fig, the number of p8-expressing target Jurkat T-cells (blue cells with red dots) is much lower in presence of a VASP-specific shRNA compared to a control (shNonsense). Evaluation of 15–20 independent optical fields in four independent experiments revealed that repression of endogenous and overexpressed VASP by VASP-specific shRNAs reduced p8 transfer to target T-cells by approximately 40% compared to a control shRNA (Fig 6C; p<0.01; n = 5). In parallel, western blot analysis revealed that shRNAs targeting VASP resulted in robust repression of both endogenous and overexpressed VASP protein (Fig 6D). Thus, VASP is not only a novel interaction partner of p8 (Fig 1) and found in protrusive structures (Fig 4B; Fig 5), but it also seems to be important for the transfer of p8 to target T-cells (Fig 6).

### Knockout of VASP delays transfer of p8 between T-cells

**Fig 7:**
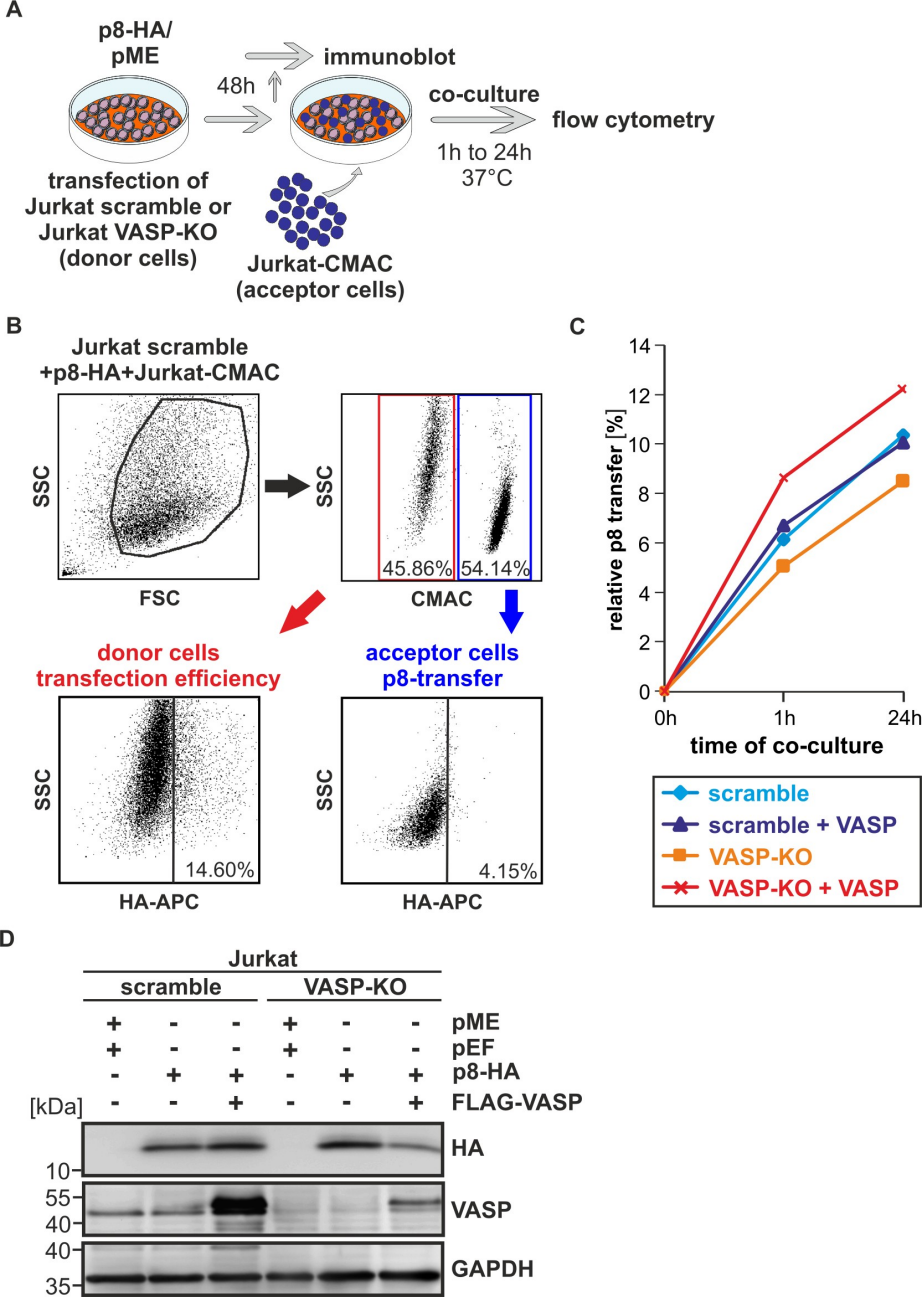
Knockout of VASP impairs p8-transfer between Jurkat T-cells. **(A)** Experimental setup. **(B-D)** Stably transduced Jurkat T-cells (guide scramble, VASP-KO) were transfected with p8-HA expression plasmids or a control plasmid (pME) and FLAG-VASP or the respective control (pEF-1α). At 48 h post transfection, cells were either **(B-C)** co-cultured with pre-stained target Jurkat T-cells (Jurkat-CMAC; ratio 1:1, 37°C) for 0 h, 1 h, or 24 h and subjected to flow cytometry, or **(D)** cells were lysed for immunoblot analysis **(B-C)** Flow cytometry. At 48 h post transfection, equal amounts of donor and acceptor cells (1x10^6^ cells each) were either directly fixed in 2% PFA and mixed (time point: 0 h), or they were co-cultured at 37°C for 1 h or 24 h before fixation. After intracellular staining using HA-specific, APC-labeled antibodies or the respective isotype-matched control antibodies, flow cytometry was performed. **(B)** Representative dot plots at 1 h post co-culture are shown. Upper left: Dot plots display the forward scatter (FSC) plotted against the side scatter (SSC) and living cells are gated (black gate). Upper right: CMAC-specific fluorescence is plotted against the SSC, which allows discrimination between CMAC-negative donor (red gate) and CMAC-positive acceptor (blue gate) cells. Lower plots: HA-specific fluorescence is plotted against the SSC and numbers represent the efficiency of transfection within the CMAC-negative donor cells (lower left) or the transfer of p8 within the CMAC-positive acceptor cells (lower right). **(C)** The relative transfer of p8 between p8-expressing CMAC-negative Jurkat donor cells (scramble, scramble and FLAG-VASP, VASP-KO, VASP-KO and FLAG-VASP) and CMAC-positive Jurkat acceptor cells is shown over time (0 h, 1 h, 24 h). The means of at least three independent experiments are shown. **(D)** Western blot analysis depicting p8-HA and VASP in stably transduced Jurkat T-cells (scramble, VASP-KO).

To confirm these findings with different methods, we made use of our recently described flow cytometry-based assay [29] and generated stable Jurkat T-cells with a knockout of VASP applying CRISPR/Cas9 technology. For this purpose, we cloned two different VASP-specific guide RNAs into the lentiviral vector system lentiCRISPRv2 [53] and transduced Jurkat T-cells either with a pool of both lentiCRISPRv2-VASP-guide1 and lentiCRISPRv2-VASP-guide2 vectors or with the unspecific control vector lentiCRISPRv2-scramble-guide [19]. After transduction and selection, western blot analysis revealed that VASP protein was knocked out at day 14 and day 28 post transduction in the newly generated cell line Jurkat VASP-KO (knockout) compared to the Jurkat scramble control (S4A Fig). Further, propidium iodide staining showed that vitality of Jurkat scramble and Jurkat VASP-KO cells was comparable to that of untreated Jurkat T-cells, while vitality in cells treated with the topoisomerase II inhibitor etoposide was significantly impaired (S4B Fig; p<0.05; n = 3). To analyze the impact of VASP on p8 transfer in this new model system, the donor cells Jurkat scramble or Jurkat VASP-KO were transfected with p8-HA or control plasmids (pME) and either supplemented with FLAG-VASP or the respective empty control plasmids. After 2 days, donor cells were co-cultured with pre-stained acceptor Jurkat T-cells (Jurkat-CMAC; ratio 1:1, 37°C) for different time points (0 h, 1 h, 24 h) and subjected to intracellular staining with HA-specific antibodies and flow cytometry, or cells were lysed for immunoblot analysis (Fig 7A). A representative dot plot shows the co-culture of Jurkat scramble cells expressing p8-HA (donor cells) with Jurkat-CMAC acceptor cells (Fig 7B). After gating on living cells based on FSC/SSC (Fig 7B, upper left, black gate), CMAC-staining (Fig 7B, upper right) allowed the discrimination between Jurkat donor cells (CMAC-negative; red gate) and Jurkat acceptor cells (CMAC-positive; blue gate). Briefly, after entering living cells, the dye CMAC gets membrane-impermeable, and it is retained in living cells for several generations, transferred to daughter cells, but not to neighboring cells [54]. Thus, use of this dye allows for a proper discrimination between p8-positive, CMAC-negative donor cells and Jurkat-CMAC acceptor cells (Fig 7B, upper right). Further gating on CMAC negative donor cells indicated that ca. 14.6% of the donor cells were expressing p8 (Fig 7B, lower left), reflecting the transfection efficiency, while ca. 4.15% of the CMAC-positive acceptor Jurkat cells received p8 after 1 h of co-culture (Fig 7B, lower right). To evaluate the relative transfer of p8 over time, co-cultures were analyzed at 0 h, 1 h, or 24 h post co-culture and the relative transfer of p8 was calculated as previously described [29]. Briefly, the relative transfer of p8 was calculated by normalizing the transfer of p8 (Jurkat-CMAC-positive acceptor cells) on the respective transfection efficiency (Jurkat-CMAC-negative donor cells), taking into account background fluorescence signals of co-cultures between acceptor cells and the respective CMAC-negative donor cell lines transfected with an empty vector plasmid (pME). As shown in Fig 7C, the relative p8-transfer in the Jurkat scramble control cells (Fig 7C, light blue) increased over time from 0% (0 h) to 6.15% (1 h) up to 10.35% (24 h) confirming earlier observations [29]. Comparison of the Jurkat scramble cells (Fig 7C, light blue; control) with Jurkat VASP-KO cells (Fig 7C, orange) revealed that knockout of VASP impaired the relative transfer of p8 to target Jurkat T-cells over time. Interestingly, overexpression of VASP in Jurkat scramble cells did not further enhance the relative p8-transfer (Fig 7C, dark blue), suggesting that the levels of endogenous VASP are sufficient to mediate p8 transfer. However, upon knockout of endogenous VASP (Fig 7C, orange), reconstitution of VASP (Fig 7C, red) led to a strong increase of the relative p8-transfer over time also exceeding the p8-transfer observed in the control cells Jurkat scramble (Fig 7C, light blue). Western blot analysis confirmed the phenotype of the VASP-scramble and VASP-KO cells and expression of p8-HA and FLAG-VASP as expected (Fig 7D). Overall, while the relative transfer of p8 sharply increases within 1 h, the increase of p8-transfer is less between 1 h and 24 h of co-culture reflected by the reduced steepness of the curves (Fig 7C). Considering that VASP is important for p8-transfer over time, but observing that the curve progression runs almost parallel between 1 h and 24 h of co-culture independent of the experimental condition, suggests that VASP may be more critical for the initial steps of p8 transfer observed between 0 h and 1 h of co-culture. Together, these data show that knockout of VASP delays p8 transfer between cells (Fig 7) supporting our previous finding that VASP is important for the transfer of p8 to target T-cells (Fig 6).

### VASP is important for membrane-recruitment of p8 and can contribute to p8-induced protrusion formation

**Fig 8:**
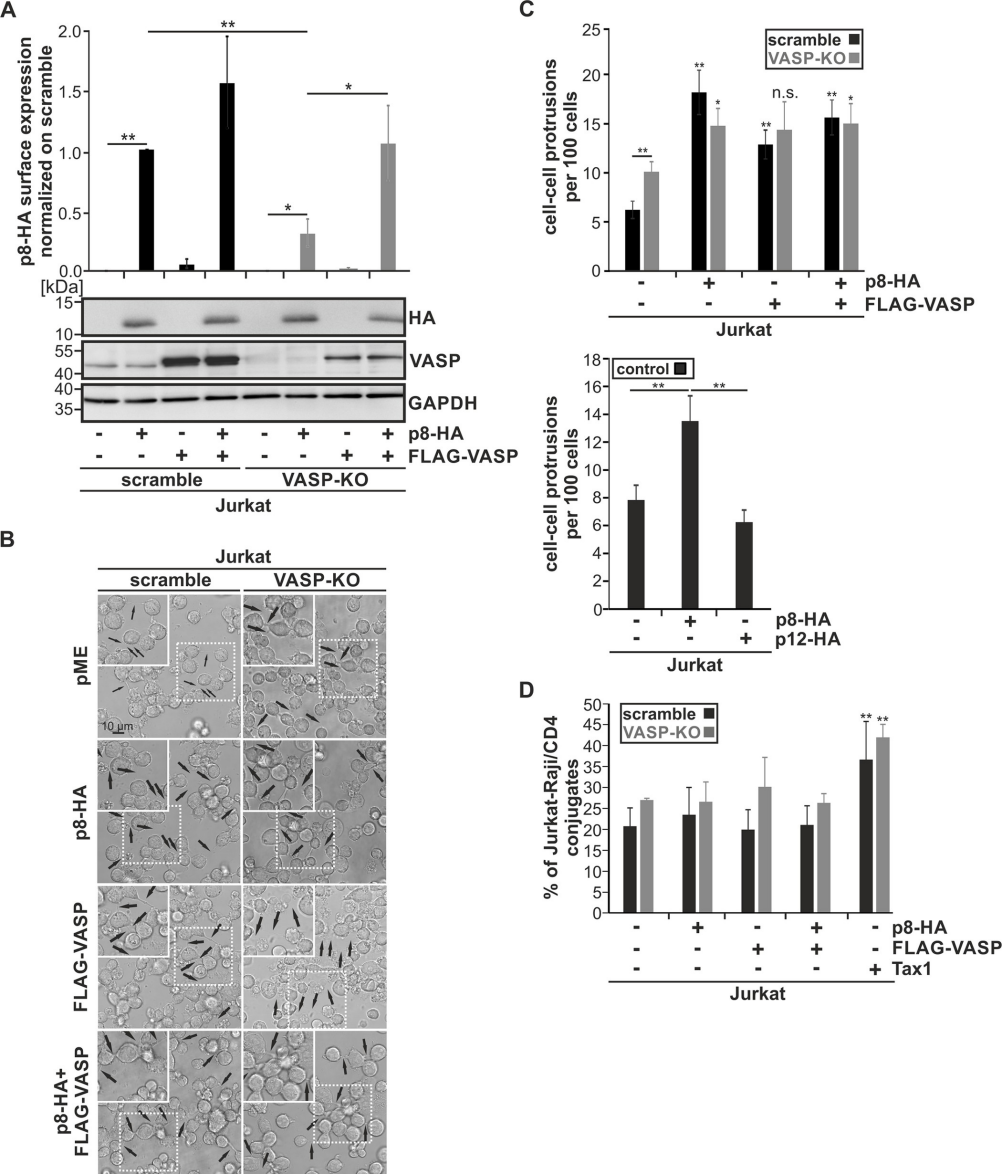
VASP is crucial for recruitment of p8 to the cell surface. **(A-D)** Jurkat scramble or Jurkat VASP-KO cells were transfected with p8-HA, FLAG-VASP or both plasmids. Cells transfected with empty vectors pME and pEF-1α served as control. All samples were replenished with the respective empty vectors to 100 μg. At 48 h post transfection, **(A)** p8 surface expression, **(B-C)** cell-cell-protrusion formation, or **(D)** cell-cell conjugate formation were analyzed in at least three independent experiments. In parallel, protein lysates were isolated and subjected to Western blot analysis. **(A)** Transfected Jurkat scramble (black bars) and VASP-KO cells (grey bars) were stained without permeabilization using HA-specific, APC-labeled antibodies or the respective isotype-matched control antibodies and flow cytometry was performed. The mean fold change p8-HA surface expression of four independent experiments was compared using Student’s test (*, p<0.05; **, p<0.01). Additionally, a representative Western blot depicting p8-HA, VASP or the housekeeping gene GAPDH is shown. **(B)** Transfected cells were cultured without fixation in micro-slides for 1 h at 37°C and analyzed with a Leica TCS SP5 confocal laser scanning microscope equipped with a 63x1.4 HCX PL APO CS oil immersion objective lens. Transmitted light is shown. Black arrows indicate cell-cell-protrusions. Insets (dashed lines) are shown as enlargement (solid lines) **(C)** Quantitative analysis of data exemplified in **(B)** in Jurkat scramble (upper panel, black bars) and VASP-KO cells upper panel, dark grey bars), and as a control, in Jurkat cells (lower panel,black bars). In the latter case, cells transfected with p12-HA expression plasmids served as control. At least 20 optical fields per experimental condition were analyzed and mean numbers of cell-cell-protrusions per 100 cells were compared using Student’s t-test (*, p<0.05; **, p<0.01; n.s., not significant). **(D)** Conjugate formation between transfected Jurkat T-cells (scramble: black bars; VASP-KO, grey bars) and co-cultured Raji/CD4^+^ B-cells was quantitated by flow cytometry. Jurkat cells transfected with the Tax-expression construct pEF1α-Tax served as positive control. After 24h, Jurkat T-cells were co-cultured with Raji/CD4^+^ B-cells (ratio 1:1) for 1 h at 37°C. Co-cultures were fixed and stained with anti-CD3-AlexaFluor700 (for Jurkat T-cells) and anti-HLA-DR-PacificBlue antibodies (for Raji/CD4^+^ B-cells) to differentiate between the two cell types. Cell-cell conjugates were identified as double-positive signals (HLA-DR^+^CD3^+^) and normalized on the total number of Jurkat T-cells. The means of three independent experiments ± standard deviation are shown and were compared using Student's t-test (**, p<0.01).

To elucidate how VASP contributes to p8 transfer, we first asked whether VASP is crucial for initial steps of p8 transfer like recruitment of p8 to the plasma membrane. Since earlier work has shown that p8 localizes to the plasma membrane and can be detected by staining the C-terminal HA-tag in non-permeabilized cells [20,29,55], we performed extracellular staining of p8-HA in Jurkat VASP-KO and Jurkat scramble control cells (Fig 8A, upper panel). Flow cytometry confirmed surface expression of p8-HA and found a moderately (1.5 fold) but not significantly enhanced surface expression of p8-HA upon overexpression of FLAG-VASP in Jurkat scramble cells (Fig 8A, upper panel). However, compared to Jurkat scramble (1.0 fold), knockout of VASP led to a significant reduction of p8-HA surface expression to 0.32 fold (Fig 8A, upper panel; p<0.05; n = 4) despite comparable amounts of total p8-HA protein expression as detected by western blot analysis (Fig 8A, lower panel). Finally, overexpression of FLAG-VASP led to a significant increase of p8-HA surface expression in VASP-KO cells compared to VASP-KO cells expressing p8-HA only (Fig 8A, upper panel; p<0.05; n = 4). Together, these data indicate that endogenous VASP is important for surface expression of p8 and suggest that VASP contributes to membrane recruitment of p8, an initial step of p8 transfer, which is in line with our data showing that knockout of VASP delays p8 transfer between cells (Fig 7). Since p8 induces protrusive structures, we wondered whether p8-induced formation of cell-cell-protrusions depends on VASP. For this purpose, we analyzed Jurkat scramble and VASP-KO cells after expression of p8-HA, FLAG-VASP and both constructs compared to controls transfected with empty vectors. Since fixation impairs formation and stability of protrusions significantly [16], a fact we also observed in this study when counting protrusions in fixed cells (Fig 5B and 5C), we analyzed living cells (Fig 8B and 8C) and controlled protein expression by western blot analysis of the same samples (S5A Fig). Analysis of transmitted light revealed that cell-cell-protrusions are detectable in Jurkat scramble and VASP-KO cells independent of overexpressing p8 or VASP (Fig 8B, black arrows). However, quantitation of results revealed the following (Fig 8C, upper panel): (1) In Jurkat scramble cells, expression of p8, VASP, or both proteins significantly enhanced the number of cell-cell protrusions to comparable levels, confirming earlier studies showing that p8 induces protrusions [15,16]. This suggests that both p8 and VASP are able to induce protrusions independent of each other. (2) Unexpectedly, knockout of VASP led to a significant enhanced numbers of cell-cell protrusions in mock-transfected cells compared to Jurkat scramble cells. Despite absence of VASP, p8 was still able to induce cell-cell protrusions (Fig 8C, upper panel, grey bars, 2-fold increase from 10 to 15 cell-cell protrusions per 100 cells), but the increase in p8-induced protrusion formation was much higher in presence of endogenous VASP (Fig 8C, upper panel, black bars, 3-fold increase from 6 to 18 cell-cell protrusions per 100 cells). Overexpression of VASP and of both p8 and VASP led to comparable numbers of cell-cell-protrusions like upon expression of p8 only. As a control, we also analyzed normal Jurkat T-cells and could confirm that p8 enhances the number of cell-cell protrusions while p12 does not (Fig 8C, lower panel) [15]. Together, these data suggest that endogenous VASP can contribute to p8-induced protrusion formation, but to a greater extent, p8 induces cell-cell protrusions independent of VASP. Finally, we asked whether VASP modulates cell-cell-aggregation and could thus enhance p8-transfer to target cells. For this purpose, Jurkat scramble and Jurkat VASP-KO cells were co-cultured with Raji/CD4^+^ B-cells and analyzed for cell-cell aggregation by flow cytometry. While overexpression of Tax led to a significant increase of Jurkat:Raji/CD4^+^ aggregates (p<0.01; n = 3) confirming earlier observations [52], neither p8 nor FLAG-VASP led to an enhancement of Jurkat:Raji/CD4^+^ aggregates (Fig 8D) despite detectable protein expression levels (S5B Fig). In summary, our data show that VASP is crucial for recruitment of p8 to the plasma membrane.

### VASP is important for p8-transfer in chronically infected T-cells

**Fig 9:**
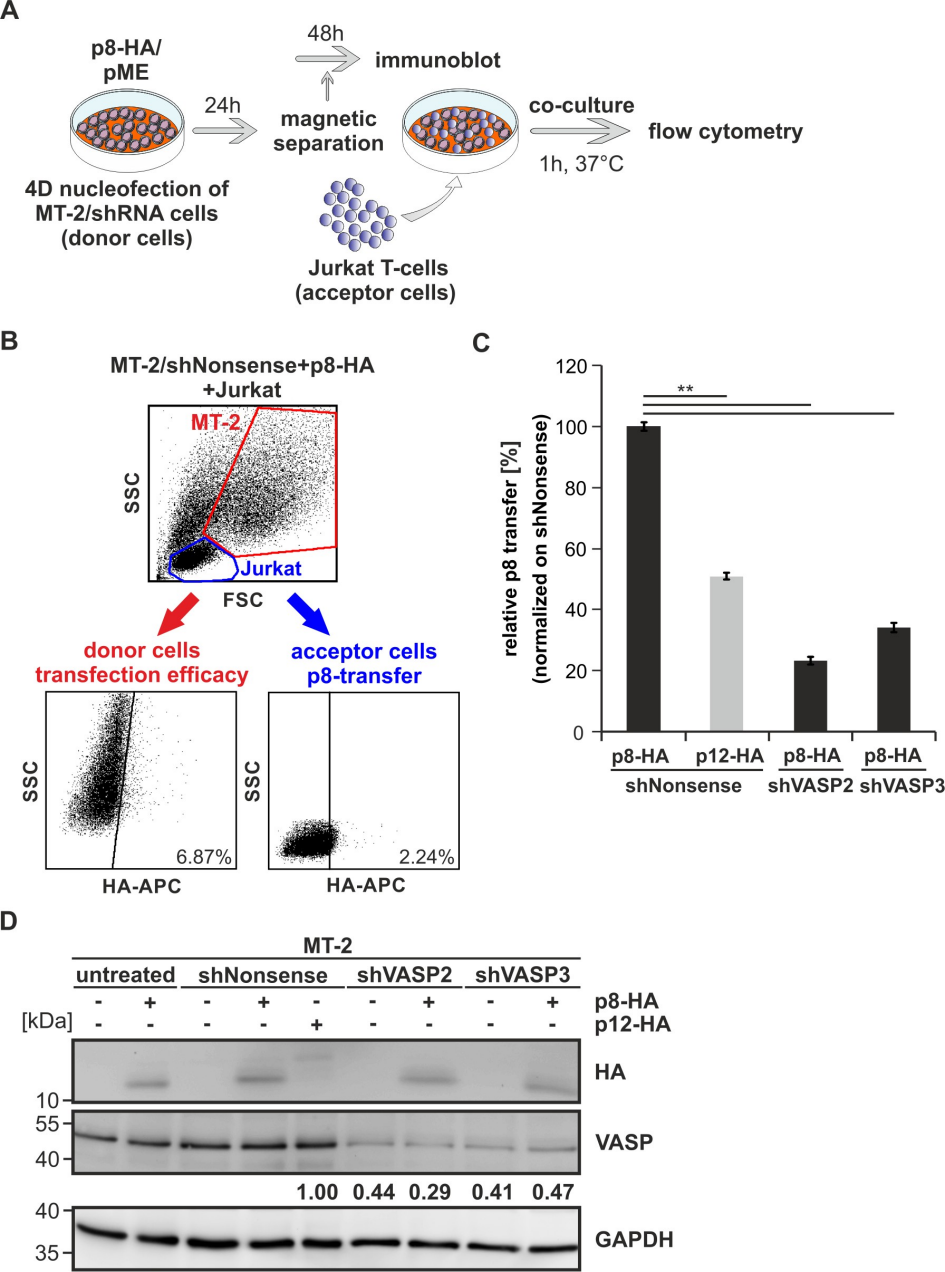
Repression of VASP impairs p8-transfer between chronically-infected MT-2 cells and uninfected T-cells. **(A)** Experimental setup. **(B-D)** Stably transduced MT-2 cells (shNonsense, shVASP2, shVASP3) were nucleofected with p8-HA expression plasmids or a control plasmid together with pMACS-LNGFR. At 24 h post transfection, transfected MT-2 cells were enriched by magnetic separation using anti-LNGFR microbeads. Separated MT-2 cells were either **(B-C)** co-cultured with target Jurkat T-cells (ratio 1:1) for 1 h at 37°C and subjected to flow cytometry or **(D)** MT-2 cells were cultured for another 48 h and lysed for western blot analysis. **(B)** Flow cytometry of co-cultures after use of primary mouse anti-HA-APC conjugated specific antibodies. Representative dot plots are shown. Upper part: Jurkat acceptor cells (blue) and MT-2 donor cells (red) were gated by forward scatter (FSC) and side scatter (SSC) analysis. Lower part: HA-APC-specific fluorescence is plotted against the SSC and numbers represent the efficiency of transfection within the MT-2 donor cells (red, left side) or the transfer of p8 within the Jurkat acceptor T-cells (blue, right side). **(C)** The relative transfer of p8 from MT-2 cell lines (shNonsense, shVASP2, shVASP3) transfected with p8-HA or p12-HA to co-cultured Jurkat T-cells was calculated and values were normalized on MT-2/shNonsense. The means of four independent experiments ± SE were compared using Student’s t-test (**, p<0.01). **(D)** Western blot analysis depicting p8-HA or p12-HA and VASP in stably transduced MT-2 cells (shNonsense, shVASP2, shVASP3). Numbers indicate densitometric analysis of VASP normalized on GAPDH and shNonsense.

To test whether VASP also plays a role in p8 transfer in chronically HTLV-1-infected T-cells, we stably transduced the HTLV-1-infected cell line MT-2 with retroviral vectors encoding VASP-specific shRNAs (shVASP2 or shVASP3), or the control shRNA shNonsense. After selection, stable transduction of MT-2 cells was monitored by flow cytometry and stable MT-2 cells were co-transfected by nucleofection with p8-HA expression plasmids or pME (control) together with a truncated version of LNGFR to enrich the number of transfected and p8-expressing cells by magnetic separation. MT-2/shNonsense cells were also transfected with p12-HA expression plasmids. After 24 h, transfected cells were enriched using LNGFR-specific microbeads and analyzed by flow cytometry. Enriched MT-2 cells were either co-cultured with acceptor Jurkat T-cells (1 h, 37°C), or they were cultured for another 48 h and subjected to protein lysis and western blot analysis (Fig 9A). A representative flow cytometry analysis of co-cultures after staining of HA shows that Jurkat T-cells (Fig 9B, upper part, blue gate, acceptor cells) were discriminated from MT-2 cells (Fig 9B, upper part, red gate, donor cells) according to their FSC and SSC. In parallel, we also discriminated Jurkat T-cells from MT-2 cells by staining for the IL-2Rα chain CD25, which is present on MT-2 cells, but not on Jurkat T-cells [56] as described previously [52], which gave comparable results. A representative dot plot shows that ca. 6.87% of transfected MT-2 cells expressed p8 (Fig 9B, lower left), while 2.24% of co-cultured Jurkat T-cells received p8 after 1 h of co-culture. Together, this confirms earlier, imaging-based work that ectopically expressed p8 is transferred between MT-2 cells and co-cultured acceptor cells [15], but extends this work by allowing a quantitative evaluation of the relative p8-transfer. Calculating the relative transfer of p8 revealed that approximately one third of co-cultured Jurkat acceptor T-cells received p8 after 1 h of co-culture with p8-expressing MT-2 donor cells (Fig 9B). In contrast, co-cultures between Jurkat T-cells resulted in ca. 6.15% of relative p8 transfer after 1 h of co-culture (Fig 7C), suggesting that HTLV-1-infected MT-2 cells are either quicker or better p8-donor cells than Jurkat T-cells. Compared to MT-2/shNonsense (100%; Fig 9C), repression of VASP in MT-2 cells resulted in a significant decrease of p8-transfer to Jurkat T-cells independent of the shRNA used by 76.9% (shVASP2) or 65.8% (shVASP3), respectively (Fig 9C; p<0.01; n = 4). Earlier work has shown that p12 (p12G29S), the precursor of p8, is impaired in being transferred between cells [15], which we could also confirm quantitatively. Compared to p8, less than half of the co-cultured cells received p12 (Fig 9C; p<0.01). Finally, we confirmed the expression and repression of VASP, p8 and p12 by western blotting (Fig 9D). While shVASP2 led to a reduction of VASP protein to 29–44%, shVASP3 reduced VASP protein expression to ca. 41–47%. Together, these data show that VASP is not only important for the transfer of p8 between Jurkat T-cells (Figs 6 and 7), but, to a greater extent, also for the transfer between chronically infected MT-2 cells and co-cultured Jurkat T-cells (Fig 9).

### Repression of VASP impairs HTLV-1 Gag cell-to-cell transfer

**Fig 10:**
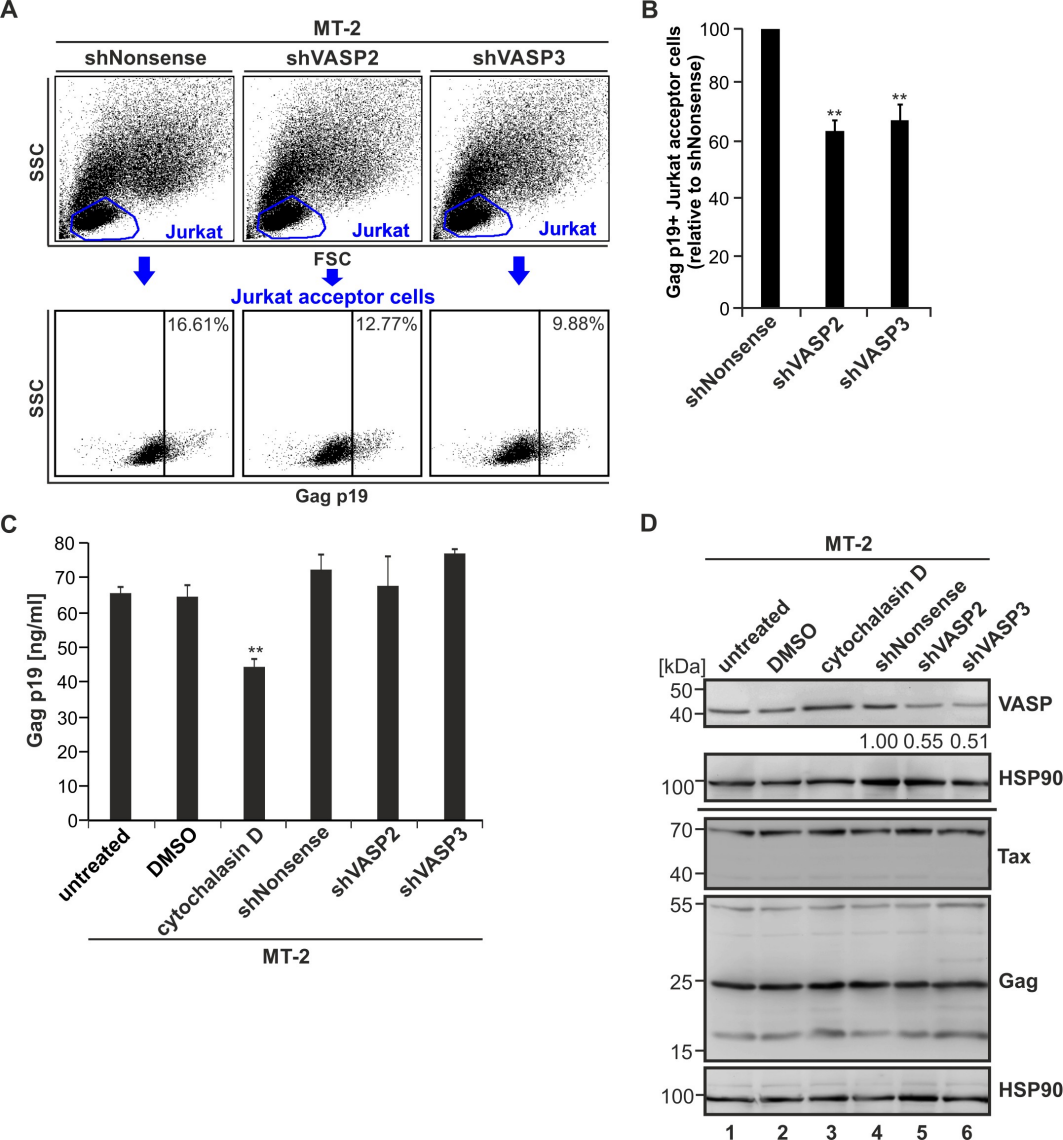
Repression of VASP impairs virus transmission to uninfected T-cells, but not Gag processing and virus release in chronically infected MT-2 cells. **(A)** Jurkat T-cells were co-cultured with stably transduced MT-2 cells (shNonsense, shVASP2, shVASP3) at a ratio of 1:1 for 1 h. The transfer of Gag p19 to Jurkat acceptor cells was measured by flow cytometry using the primary antibodies mouse anti-Gag p19 and the secondary antibodies anti-mouse Alexa Fluor 647. Upper part: Jurkat acceptor cells were gated by forward scatter (FSC) and side scatter (SSC) analysis; lower part: Gag p19-specific fluorescence (indicated by numbers) in Jurkat acceptor T-cells. **(B)** The amount of Gag p19 positive Jurkat acceptor cells normalized on the MT-2/shNonsense control cells of four independent experiments ± SE is depicted and was compared using Student’s t-test (**, p<0.01). **(C)** The amount of Gag p19 protein in the supernatant of transduced MT-2 cell lines was assessed by Gag p19 ELISA. MT-2 cells treated with 5 μM cytochalasin D, an inhibitor of actin-polymerization, in comparison to the DMSO solvent control served as positive control for an impaired Gag p19 release. The mean of four independent experiments ± SE is depicted and was compared using Student’s t-test (**, p<0.01). **(D)** Western blot analysis depicting VASP, Tax and Gag p55 precursor and processed Gag p19 matrix protein in stably transduced MT-2 cells (shNonsense, shVASP2, shVASP3) and the indicated controls. Numbers indicate densitometric analysis of VASP normalized on Hsp90 and shNonsense.

Since VASP is crucial for actin filament elongation [38] and, as this work shows, for the transfer of p8 to the cell surface and to co-cultured T-cells (Figs 6–9), we asked whether VASP also affects cell-to-cell transfer of HTLV-1 Gag, and thus, HTLV-1 transmissions, which occurs via close cell-cell contacts at the virological synapse [14,17] or via cellular protrusions that are induced by p8 [15]. To study the impact of VASP on HTLV-1 cell-to-cell transmission, we performed co-culture assays between chronically HTLV-1-infected MT-2 cells and uninfected Jurkat acceptor T-cells (ratio 1:1; 1 h, 37°C) and stained the cells for Gag (using anti-Gag p19 antibodies) and for CD25 as previously described [52]. Briefly, stable cell lines MT-2/shNonsense, MT-2/shVASP2, and MT-2/shVASP3 were used as donor cells. Flow cytometry was performed to discriminate cells according to their FSC and SSC (Fig 10A, upper panels) and based on CD25-staining, which gave similar results. After 1 h of co-culture with MT-2/shNonsense donor cells, ca. 16.61% of co-cultured Jurkat T-cells were Gag p19-positive, while only 12.77% or 9.88% of Jurkat acceptor cells were positive upon co-culture with MT-2/shVASP2 or MT-2/shVASP3, respectively (Fig 10A, lower panels). Summing up several independent experiments revealed that repression of VASP in MT-2 cells led to a significant reduction of HTLV-1 Gag transfer to target Jurkat cells by nearly 40% independent of the shRNA used to target VASP (Fig 10B; p<0.01; n = 4). Despite a significant impact on HTLV-1 Gag cell-to-cell transfer, repression of VASP did not affect release of Gag p19 in chronically-infected MT-2 cells compared to control cells that were treated with cytochalasin D, an inhibitor of actin polymerization (Fig 10C; p<0.01; n = 4). Finally, we verified the knockdown of VASP in MT-2 cells by western blot (Fig 9D). While protein expression of VASP was reduced to ca. 55% (shVASP2) or 51% (shVASP3), expression of Tax or of cell-associated Gag protein were not impaired compared to the control cell line MT-2/shNonsense. Processing of Gag from the precursor Gag p55 did also not differ between the different shRNA-carrying cell lines (Fig 10D) confirming results from Gag p19 ELISA (Fig 10C). Overall, our data show that VASP is not only a novel interaction partner of p8 and important for p8 transfer between cells, but VASP also affects Gag transfer between chronically-infected T-cells and acceptor T-cells, while release of virus-like particles is unaffected.

**Fig 11:**
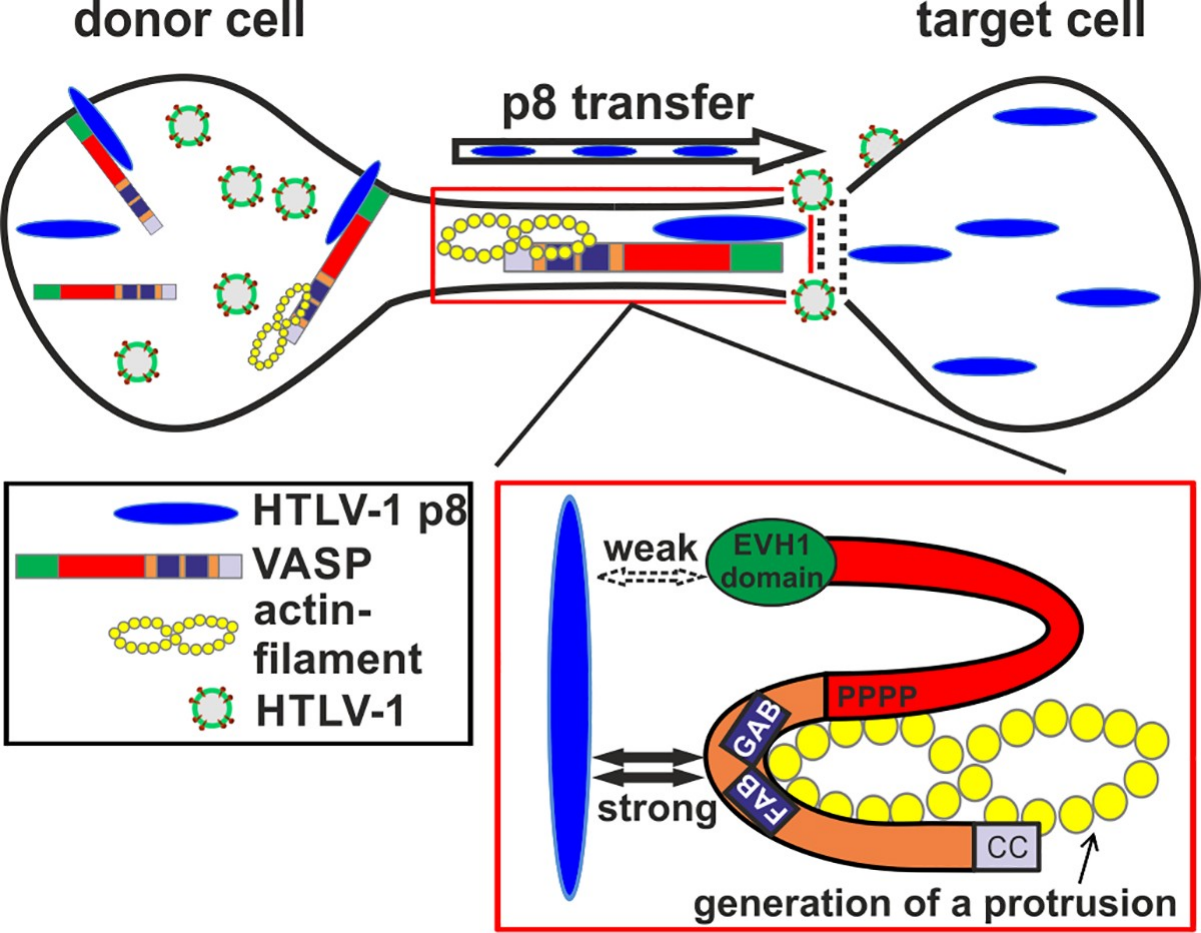
Model of the VASP:p8 interaction and its role in p8 transfer and HTLV-1 transmission. Our data suggest a weak interaction between the EVH1 domain of VASP and p8, which may be important for the recruitment of VASP, and a strong interaction of the G- and F-actin binding site of VASP with p8. Transfer of p8 and of Gag p19, and thus, presumably of HTLV-1 to target cells depends on VASP and may be driven by formation of a VASP- and actin-containing cellular conduit.

Together, our data propose a model showing an important role of VASP in HTLV-1 cell-to-cell transmission (Fig 11). Mechanistically, transfer of p8 to target cells may be driven by VASP-dependent recruitment of p8 to the cell surface and to a lesser extent, by formation of a VASP- and actin-containing cellular conduit. Our data suggest a weak interaction between the EVH1 domain of VASP and p8, which may be important for the recruitment of VASP, and a stronger interaction of the G- and F-actin binding site of VASP with p8 (Fig 11). Thus, we identified VASP as a novel interaction partner of p8 which is not only crucial for p8 transfer to target cells, but presumably also for HTLV-1 transmission.

## Discussion

HTLV-1 establishes persistent infections eventually leading to the development of severe incurable diseases in about 10% of infected carriers [9]. The accessory protein p8 and its precursor p12 encoded from orf I in the HTLV-1 pX region are thought to play a crucial role in establishing viral persistence [24,25,27,32]. As a distinctive feature, the p8 protein enhances conduit and tunneling nanotube (TNT) formation in infected cells, is transferred to target T-cells, and fosters HTLV-1 transmission [15,16,29]. However, it was unclear how p8 is transported to other cells.

In this study, we identified vasodilator-stimulated phosphoprotein (VASP) as a novel interaction partner of p8, which is crucial for the transport of p8 to other cells. Interestingly, we found that VASP is also important for transfer of the HTLV-1 Gag protein between cells, and thus, presumably for HTLV-1 cell-to-cell transmission. VASP, a member of the Ena/VASP protein family, prevents actin-filaments from capping thereby promoting filament elongation [38]. Starting from bioinformatics, we identified the proline-rich sequence stretch p8 _24_LFLLFLPLFFSLPLLLSPSLPL_45_ as a potential motif mediating interactions with the VASP EVH1 domain. Putative interactions of p8 with VASP were confirmed by co-immunoprecipitation and immunofluorescence in different cell types. Despite its localization in lipid rafts [20], our data suggest that the VASP:p8 interaction is specific as it was also detectable in the presence of the detergent N-octyl-β-D-glucoside, which solubilizes membrane proteins [44]. Mutational studies revealed that the EVH1-domain of VASP is necessary, but not sufficient for the interaction with p8. Deletion of the EVH1-domain within VASP significantly diminished precipitation of p8, however, expression of the EVH1-domain alone only co-precipitated very low amounts of p8. This suggests that p8 encodes a low-affinity EVH1 binding motif, which may allow for transient binding of p8 to VASP. This is supported by the notion that the EVH1 domain usually binds motifs with a higher proline content like the FPPPP motif, which is found in the cellular proteins Zyxin and Vinculin and the *Listeria monocytogenes* protein ActA [37,57,58]. Since the EVH1-domain of VASP alone is not sufficient to efficiently precipitate p8, this also suggests that the EVH1 domain may just be required for efficient recruiting of VASP, or for the resolution of an unfavorable VASP conformation. Moreover, both the EVH1 and the EVH2 domains of VASP are required for VASP localization to focal adhesions [48], which may be a pre-requisite for the interaction with p8, which is also localized in the cytoplasm and at the plasma membrane in cells [20]. Since the G- and F-actin binding domains located in the EVH2 domain of VASP are required for the interaction with p8, it cannot be fully excluded that the VASP:p8 interaction depends on actin. However, it is also conceivable that actin is only required for positioning of VASP at the plasma membrane [48], thus allowing interactions between VASP and p8. Our data identifying the p8 mimicking peptide p8(26–37) to block the VASP:p8 interaction harbor the potential to develop therapeutic modified peptides, which may also be used and tested in living cells for their capacity to inhibit the transfer of p8 in future work.

VASP had been originally identified as a major substrate of cAMP-dependent (PKA) and cGMP-dependent protein kinase (PKG) in platelets [33]. Up to now, several other kinases have been identified to differentially phosphorylate VASP, and thus, to control modulation of the actin cytoskeleton [59]. Interestingly, we could show that intracellular cAMP levels are upregulated in chronically HTLV-1 infected T-cells [60], and it is conceivable that phosphorylated VASP-species are present in these cells. Since not only the EVH1 domain, but also phosphorylation of Ser153 are required for VASP localization to focal adhesions and for the affinity of VASP to F-Actin [61,62], it cannot be excluded yet that phosphorylation of VASP is important for interacting with p8, or even for p8 and HTLV-1 transfer.

Our work confirms earlier findings showing transfer of p8 to Jurkat T-cells [15,16,29]. Beyond, we provide evidence that VASP is important for the transfer of p8 to target T-cells since we detected VASP-containing protrusive structures that also contained p8. Knockdown and knockout experiments underlined the importance of VASP for the transfer of p8 to target Jurkat T-cells since repression of both endogenous and overexpressed VASP reduced the number of p8-positive target T-cells and delayed the transfer of p8 between cells. The latter suggests that VASP is important for early events of p8 transfer like membrane recruitment of p8, which is underlined by our data showing VASP-dependent expression of p8 at the cell surface. Furthermore, those domains in VASP that are required for the interaction with p8 are also required for VASP localization to focal adhesions [38]. We could confirm that p8 induces cell-cell protrusions and we found that p8 enhances cell-cell-protrusion formation more strongly in cells expressing endogenous VASP than in VASP-KO cells. Although our data show that VASP does not further enhance the number of p8-induced cell-cell protrusions, it cannot be excluded that protrusions containing VASP and p8 in co-localization are more stable, and thus, could contribute to transfer of p8. It has been proposed earlier that HTLV-1 benefits from p8 transfer to other cells. While the precursor p12 modulates T-cell activation, p8 is supposed to dampen T-cell receptor signaling and to anergize T-cells prior to infection [23]. Beyond, it was suggested that upon transfer of p8 to CD8^+^ T-cells or NK-cells, it may interfere with antiviral responses [15,30]. However, it has not been formally proven yet whether p8, and not only its precursor p12, is able to dampen T-cell or antiviral responses. Furthermore, it is still not settled to which target cells p8 is transported, and whether this also occurs *in vivo*.

We found that repression of VASP leads to a significant reduction of HTLV-1 Gag transfer between cells, suggesting that HTLV-1 cell-to-cell transmission depends on VASP. Yet, the mechanism of VASP-dependent Gag transfer has to be elucidated, e.g. whether a mini VS is formed at the tip of cellular protrusions, or whether viral biofilm is transferred along VASP containing protrusions leading to productive infection of target cells. Further, it is conceivable that VASP may also be crucial for recruitment of Gag to the plasma membrane, similar to the findings we obtained for p8. Thus, it cannot be excluded that transfer of p8 and Gag occur independently. Hitherto, it is unclear whether VASP plays also a direct role in p8-induced HTLV-1 transmission and whether VASP is crucial for Gag transfer and virus transmission in related viruses among the Primate T-lymphotropic virus (PTLV) family. This is of special interest since recent work has shown that one or more accessory orfs including p12 are lacking in some of the PTLV-1 strains including simian T-lymphotropic virus (STLV) and some strains of HTLV-1 (HTLV-1b and HTLV-1c) [63]. Interestingly, not only the HTLV-1-encoded protein p8, but also other microbial effectors exploit interactions with VASP to promote infectivity. *Listeria monocytogenes*, a gram positive, motile bacterium causing listeriosis, spreads via comet tails that are composed of actin filaments. The bacterial protein ActA interacts with VASP to foster bacterial spreading [39,58,62]. Contrary, during infections with the intracellular bacterial pathogens *Shigella spp*., which cause diarrheal disease in humans, VASP and the related protein Ena/VASP-like (EVL) limit bacterial spread [61]. Interestingly, VASP activity is also required for *Coxiella burnetii* growth in human macrophages [64]. Among viruses, Vaccinia uses actin-based motility for virion movement in host cells, and recruits the Ena/VASP family members N-WASP and VASP to the site of actin assembly [65,66].

Not only p8, but also its precursor p12 are thought to be important for establishing efficient persistent infections, since both proteins are required to provide resistance to killing by cytotoxic T lymphocytes, and thus, to establish productive infections in Rhesus macaques [32]. However, sequence analyses of HTLV-1-infected subjects found that ca. 5% of the subjects analyzed do not carry functional p12 sequences, suggesting that transmission and maintenance of HTLV-1-infections can also occur independent of p12 [67]. Earlier work has shown that, p12 protects HTLV-1-infected cells from NK cell-mediated cytotoxicity [68]. Based on *in vitro* data, p8 is of importance for the productive infection of monocytes [32]. We found that transfer of p12 is present but significantly less compared to p8 confirming earlier observations [15]. While p8 localizes to the cytoplasm and is recruited to lipid rafts and the immunological synapse upon T-cell receptor ligation [20], p12 is mainly localized to the ER and Golgi apparatus [21,22]. Thus, p12 unlike p8 may not get in direct contact with VASP, which is predominantly located at the cell periphery [48]. It is also conceivable that residual low levels of endogenous VASP are sufficient to mediate p8 transfer or additional cellular proteins may be recruited to allow for the formation of protrusions and for p8 transfer to other cells. For instance, it remains to be determined whether other proteins than VASP containing EVH1 domains like WASP [36] also interacts with p8. It is conceivable that p8 makes use of multiple interactions during transfer to other cells and the composition of a “transport of p8 complex” (TOPC) remains to be elucidated. We could recently show that the Tax-inducible actin-bundling protein Fascin is crucial for HTLV-1 cell-to-cell transmission and that Fascin is also localized in protrusive structures between cells [51,52]. Considering that VASP and its relative Ena cooperate with Fascin in the formation of filopodial structures [69–71], Fascin may also contribute to p8 transfer.

In summary, our work identifying VASP as a novel interaction partner of p8 is the first to shed light on the molecular mechanisms of p8 transport between cells and it uncovers VASP as a potential target to counteract HTLV-1 cell-to-cell transmission.

## Materials and methods

### Cell culture

The CD4^+^ T-cell line Jurkat (from acute lymphoblastic leukemia; ATCC, LGC Standards GmbH, Wesel, Germany) was cultured in RPMI 1640 (GIBCO, Life Technologies, Darmstadt, Germany) supplemented with 45% Panserin 401 (PAN-Biotech, Aidenbach, Germany), 10% fetal calf serum (FCS), L-glutamine (0.35 g/l) and penicillin/streptomycin (0.12 g/l each). The HTLV-1 *in vitro* transformed CD4^+^ T-cell line MT-2 [72] (kindly provided by Ralph Grassmann, deceased, FAU, Erlangen, Germany) was cultured in RPMI 1640 containing 10% FCS, L-glutamine and penicillin/streptomycin. 293T cells (kindly provided by Ralph Grassmann, deceased, FAU, Erlangen, Germany) were cultured in DMEM (GIBCO, Life Technologies), 10% FCS, L-glutamine and penicillin/streptomycin. The human Epstein-Barr virus (EBV)-positive B-cell line Raji containing the CD4 surface receptor (Raji/CD4^+^) was a kind gift from Vineet N. Kewal Ramani (NIH, Frederick,Maryland, USA) and was cultured in RPMI 1640M, Panserin, 10% FCS, L-glutamine and Pen/Strep containing 500 μg/ml geneticin [73].

### Plasmids

Plasmids p8-HA (pME18S-p12^I^-Δ29), p12-HA (pME18S-p12^I^-G29S) and the respective control pME (pME18S) were kindly provided by Genoveffa Franchini and have been described earlier [20,23,24,55]. Expression plasmids FLAG-VASP and the respective mutants FLAG-VASP-EVH1, FLAG-VASPΔEVH1 (PP-EVH2), FLAG-VASPΔPP, FLAG-VASPΔGAB (ΔA), FLAG-VASPΔFAB (ΔB), FLAG-VASPΔD, FLAG-VASPΔC and FLAG-EVH2 were kindly provided by Robert Grosse [48]. Briefly, derivatives of human VASP were expressed as follows: VASP-EVH1, expressing codons 1–113; VASPΔEVH1, missing codons 1–111; VASPΔPP, missing codons 114–224; VASP-EVH2, expressing codons 225–380; in VASPΔGAB, VASPΔFAB, and VASPΔD codons 225–245, 258–276 and 300–315, respectively, are replaced by a *Not*I site, introducing three (ΔGAB, ΔD) or two (ΔFAB) alanine codons [48]; VASPΔC lacks residues 324–380, encodes 20 different amino acids and a stop codon. pEF-1α (Life Technologies) served as control. The expression plasmid FLAG-N-WASP was kindly provided by Hitomi Mimuro (University of Tokyo, Japan) [74]. All constructs were checked for integrity by automated sequencing. The expression plasmid pMACS-LNGFR was obtained from Miltenyi Biotec (Bergisch-Gladbach, Germany). The retroviral vector pSiren-RetroQ-IRES-EGFP-shNonsense (shNonsense) carrying an unspecifc small hairpin (sh) RNA (kind gift from Frank Neipel) and the lentiCRISPRv2 vector system (kind gift from Feng Zhang, Addgene plasmid #52961) have been described earlier [53,75]. The HIV-1 gag-pol expression vector psPAX2 and pMD2.G encoding vesicular stomatitis virus glycoprotein (VSV-G) were kind gifts from Didier Trono (Addgene plasmids #12260 and #12259). The expression plasmid pEF1α-Tax encoding HTLV-1 Tax-1 has been described earlier [76].

### Transient transfections

293T cells were seeded one day prior to transfection at 5x10^5^ cells per six-well. Cells were transfected using *GeneJuice* reagent (Merck Millipore, Darmstadt, Germany) according to the manufacturer’s protocol using a total amount of 2 μg DNA. Transient transfections of Jurkat T-cells by electroporation were performed as described earlier [50]. Briefly, 1x10^7^ Jurkat T-cells were transfected using a total amount of 100 μg plasmid DNA. 3 x 10^6^ HTLV-1-infected MT-2 cells stably carrying shNonsense, shVASP2 or shVASP3 were transfected with either p8-HA, p12-HA or the control plasmid pME (10 μg each) together with 1 μg pMACS-LNGFR using the 4D nucleofector (LONZA; solution SG, programme DN100). After 24 h, transfected cells were enriched using magnetic separation (*see* Magnetic separation and co-culture assays).

### Co-immunoprecipitation (IP) and immunoblot

At 48 h post transfection, 293T or Jurkat T-cells were washed in phosphate-buffered saline (PBS) and lysed. MT-2 cells were enriched with LNGFR-specific microbeads at 24 h post nucleofection (*see* Magnetic separation and co-culture assays) and cultured for another 48 h at 37°C prior to lysis. TNE lysis buffer contained 150 mM NaCl, 10 mM Tris/HCl (pH 7.0), 10 mM EDTA, 1% Triton X-100, 2 mM DTT supplemented with protease inhibitors leupeptin, aprotinin (20 μg/ml each) and 1 mM phenyl-methylsulfonyl fluoride (PMSF; 1 mM). After repeated freeze-and-thaw cycles in liquid nitrogen, lysates were centrifuged at 14.000 rpm (15 min, 4°C). Supernatants containing cellular proteins were separated: 10% were taken as input control and the rest was subjected to co-immunoprecipitation (IP). For this purpose, protein G-coupled magnetic beads (Dynabeads; Life Technologies) were rotated 10 min with 5 μg anti-FLAG (M2; Sigma Aldrich, Darmstadt, Germany) or 2 μg anti-VASP antibodies (Enzo Life Sciences, Lörrach, Germany) or isotype control antibodies (Life Technologies) at 20°C. Then, lysates were added and rotated again for 1 h at 20°C. Samples were washed in TNE lysis buffer and eluted in 50 mM glycine (pH 2.6) and sodium dodecyl sulfate (SDS) loading dye (10 mM Tris/HCl (pH 6.8), 10% glycerine, 2% SDS, 0.1% bromphenole blue, 5% β-mercaptoethanol) (10 min, 95°C). Input lysates were denatured for 10 min at 95°C in SDS loading dye, subjected to SDS-polyacrylamide gel electrophoresis (SDS-PAGE) and transferred to Nitrocellulose membranes (Whatman, Protran, Whatman GmbH, Dassel, Germany) using standard techniques. Membranes were probed with the following antibodies: rat monoclonal anti-HA-Peroxidase clone 3F10 (Roche, Mannheim, Germany) in immunoblots and rabbit polyclonal HA.11 clone 16B12 (BioLegend, San Diego, CA, USA) for IP, anti-FLAG (M2; Sigma Aldrich), anti-VASP (Enzo Life Sciences, Lörrach, Germany), anti-β-actin (ACTB; Sigma Aldrich), anti-GAPDH (GenScript, Piscataway, NJ, USA), or anti-Hsp90 (Santa Cruz Biotechnology Inc., Dallas, Texas, USA). Secondary antibodies were conjugated with horseradish peroxidase (HRP; GE Healthcare, Little Chalfont, UK) and peroxidase activity was detected by enhanced chemoluminescence using a Kodak Image Station 4000MM PRO camera (Kodak), Fujifilm LAS-1000 Intelligent Dark Box (Fujifilm), or INTAS Advanced Fluoreszenz und ECL Imager (INTAS Science Imaging Instruments, Göttingen, Germany). At least three independent experiments were performed. Intensities of specific bands were quantitated using Advanced Image Data Analyser (AIDA Version 4.22.034, Raytest Isotopenmessgeräte GmbH, Straubenhardt, Germany). The binding of p8-HA to FLAG-VASP after IP was calculated by dividing the band intensities of p8-HA (IP) by the quotient [FLAG (IN)/β-Actin (IN)] and normalized on binding to wildtype FLAG-VASP.

### Computational analyzes and peptide design

EVH1-domain-binding patterns were defined based on the sequence preferences observed for VASP EVH1 domain ligands in an experimental peptide library scan [43]. The resulting pattern [FLWY]-[FP]-{WY}-[AFGHILNPSTV]-[FLPWY] was subsequently used in the forward and reverse orientation to identify candidate EVH1-binding motifs in the HTLV-1 p8 protein sequence. For the fine mapping of the interaction site, 4 peptides were derived from the p8 sequence: p8(21–32): SNLLFLLFLPLF, p8(26–37): LLFLPLFFSLPL, p8(29–40): SLPLFFSLPLLLS (one additional serine was added at the N-terminus to increase the solubility of the peptide) and p8(36–48): PLLLSPSLPLTMR. The peptide p8(1–12): LLLRPPPAPSLL was designed as a negative control. In this peptide, the original cysteine residue was changed into serine in order to avoid the possibility of peptide dimerization through the formation of disulfide bonds. To enhance solubility, all peptides were synthesized without blocking groups at the N- or C-terminus and purified to a purity > 95% by JPT (Berlin, Germany).

### Peptide blocking experiments

293T cells were transfected with expression plasmids for p8-HA or FLAG-VASP (1 μg each). After 48 h, cells were lysed, and 10% of the lysates were taken as input. Peptides (2 mg/ml, ca. 1.4 mM) were freshly dissolved in DMSO and lysates from p8- and VASP-expressing cells were individually incubated with a 10-fold excess of peptides (1 h, 20°C) with constant agitation. The excess of each peptide was estimated according to the Michaelis-Menten kinetics [77]. To estimate the Michaelis-Menten constant K_M_, the quotients of the estimated dissociation constants (based on the related peptide ActA; [43]) and the molarities of the respective peptide were calculated. Saturation of each peptide was estimated to occur at a 10-fold excess value of the obtained constant K_M_. Afterwards pre-incubated lysates were co-incubated for 1.5 h. Co-immunoprecipitations were performed as described above (*see* Co-immunoprecipitation (IP) and immunoblot).

### Magnetic separation and co-culture assays

To enrich the amount of transfected cells, 1x10^7^ Jurkat T-cells were co-transfected with expression plasmids p8-HA (35 μg) and pMACS-LNGFR (10 μg). Depending on the experimental setup, FLAG-VASP (35 μg) or pEF (mock; 35 μg) and 20 μg of plasmids encoding shRNAs targeting VASP, or control shRNAs (shNonsense) were co-transfected. To allow enrichment of transfected MT-2 cells, 3x10^6^ cells were co-transfected with either p8-HA, p12-HA or the control plasmid pME (10 μg each) together with 1 μg pMACS-LNGFR. After 48 h (Jurkat) or 24 h (MT-2), cells were washed with PBS (without Ca^2+^ and Mg^2+^) and incubated with anti-CD271 (LNGFR) MicroBeads (Miltenyi Biotec) for 15 min (4°C). Labeled cells were separated using MACS LS columns (Miltenyi Biotec) on a MidiMACSTM Separator (Miltenyi Biotec). The percentage of LNGFR positive cells, stained with anti LNGFR-PE conjugated antibodies (ME20.4-1.H4; 1:10; Miltenyi Biotec), was determined with the BD LSRII flow cytometer (BD Biosciences, Heidelberg, Germany) before and after magnetic separation. Purified Jurkat T-cells were either directly subjected to immunofluorescence analysis or co-cultured with acceptor Jurkat T-cells (ratio 1:1) pre-stained with CellTracker Blue CMAC Dye (CMAC; Molecular Probes, Karlsruhe, Germany) on poly-L-lysine coated glass slides for 1 h at 37°C. Purified MT-2 cells were either co-cultured with acceptor Jurkat T-cells (ratio 1:1) for 1 h at 37°C, or MT-2 cells were cultured for another 48 h and subjected to protein lysis.

### Immunofluorescence and confocal laser scanning microscopy

All images were acquired using a Leica TCS SP5 confocal laser scanning microscope equipped with a 63×1.4 HCX PL APO CS oil immersion objective lens. Images were analyzed and signal intensities at chosen regions of interest (ROI) were quantified using LAS AF software (Leica, Wetzlar, Germany).

#### Subcellular localization of p8 and VASP in 293T and Jurkat T-cells

293T cells (3x10^5^ cells/experiment) were seeded on coverslips 24 h prior to transfection with expression plasmids p8-HA (1.5 μg) and FLAG-VASP (0.5 μg). Jurkat T-cells were transfected with expression plasmids p8-HA (25 μg) and FLAG-VASP (25 μg). At 48 h post transfection, Jurkat T-cells were spotted on epoxy-resin coated glass slides and both 293T and Jurkat T-cells were fixed with 2% para-formaldehyde (PFA; 60 min, 20°C). Cells were washed three times with PBS/ 0.01% Tween and permeabilized with 0.2% Triton X-100 (20 min, 4°C). After three washing steps, unspecific binding was prevented by 5% FCS/ 1% BSA in PBS (1 h, 20°C). Cells were stained with primary antibodies mouse anti-FLAG (M2, 1:500, Sigma-Aldrich, Darmstadt, Germany) and rabbit anti-HA (BioLegend; 1:100, 1 h, 20°C) followed by secondary antibodies anti-mouse AlexaFluor 488 or anti-mouse AlexaFluor 350, and anti-rabbit AlexaFluor 647 (1:200 each, Life Technologies, 30 min, 20°C). In some experiments, cells were co-stained with anti-CD98-FITC (BD Bioscience, Heidelberg, Germany, 30 min, 20°C) in PBS containing 0.5% saponin and 1% BSA. Slides were covered with *ProLong Gold antifade reagent with or without DAPI* (Molecular Probes) and analyzed by confocal laser scanning microscopy.

#### Proximity ligation assay

293T cells (3x10^5^ cells) were seeded on coverslips 24 h prior to transfection with expression plasmids p8-HA (1 μg) and FLAG-VASP (1 μg) or FLAG-N-WASP (1 μg). At 48 h post transfection, proximity ligation assays using *Duolink In Situ Red Starter Kit Mouse/Rabbit* (Sigma-Aldrich) were performed according to the manufacturer’s instructions. Briefly, cells were fixed with 2% PFA (60 min, 20°C). Next, unspecific binding was prevented by 5% FCS/1% BSA in PBS (1 h, 20°C) and cells were incubated with either one or both of the primary antibodies mouse anti-FLAG (M2, 1:500, Sigma-Aldrich) and rabbit anti-HA (1:100, BioLegend) or the respective isotype controls (1 h, 20°C). Subsequently, cells were incubated with anti-mouse and anti-rabbit secondary antibodies conjugated with oligonucleotides (1 h, 37°C). After ligation (30 min, 37°C) and amplification (100 min, 37°C), slides were covered with *ProLong Gold antifade reagent with DAPI* and analyzed by confocal laser scanning microscopy.

#### p8-transfer to Jurkat T-cells

1x10^6^ Jurkat T-cells were pre-stained with 0.5 μM *Calcein-AM* (Life Technologies) for 30 min at 37°C and washed five times with PBS. Subsequently, pre-stained Jurkat T-cells were co-cultured with 1x10^6^ of 1x10^7^ Jurkat T-cells that had been transfected with expression plasmids p8-HA (45 μg), FLAG-VASP (45 μg), and pMACS-LNGFR (10 μg) 48 h earlier. Co-cultures of Calcein-stained acceptor Jurkat T-cells and magnetically-enriched donor cells (*see* Magnetic separation and co-culture assays) were incubated on poly-L-lysine-coated coverslips for 30 min at 37°C. Afterwards, cells were fixed and stained using primary antibodies mouse anti-FLAG and rabbit anti-HA followed by secondary antibodies anti-mouse AlexaFluor 350 and anti-rabbit AlexaFluor 647 as described above. Slides were covered with *ProLong Gold antifade reagent with DAPI* and analyzed by confocal laser scanning microscopy. Protrusions (n = 59) were counted manually in fixed cells and inspected for expression of VASP-FLAG, p8-HA, or both proteins.

#### Quantitation of p8-transfer to target Jurkat T-cells

Co-cultures of p8- and VASP expressing donor Jurkat T-cells with acceptor Jurkat T-cells pre-stained with CMAC (*see* Magnetic separation and co-culture assays) were fixed in 2% PFA and stained using primary antibodies rabbit anti-HA and secondary antibodies anti-rabbit AlexaFluor 647 as described above. Slides were covered with *ProLong Gold antifade reagent* (without DAPI) and analyzed by confocal microscopy. The number of cells expressing p8 within the acceptor Jurkat T-cells was counted in 15–20 optical fields per experimental condition in four independent experiments. Numbers were normalized on the amount of p8-expressing donor cells. In total, ca. 6022 p8-expressing donor cells and 539 p8-expressing acceptor cells were analyzed.

#### Quantitation of protrusion formation

1x10^7^ stably transduced Jurkat T-cells (Jurkat scramble or VASP-KO, *see* Knockout of VASP by CRISPR/Cas9) were transfected with 50 μg p8-HA, 50 μg VASP-FLAG or both p8-HA and VASP-FLAG. In parallel, normal Jurkat T-cells were transfected with 50 μg p8-HA or 50 μg p12-HA. In all experiments, cells transfected with empty vectors pME and pEF-1α served as control. All samples were replenished with the respective empty vectors to 100 μg. At 48 h post transfection, 2.5x10^5^ cells were cultured in *μ-Slide 8 Well* plates (micro-Slide with 8 wells for cell culture & immunofluorescence; ibidi GmbH, Gräfelfing, Germany) in Hank’s Balanced Salt Solution for 1 h at 37°C. Living cells were inspected by imaging analysis and cell-cell protrusions were counted manually. At least 20 optical fields were analyzed in two independent experiments, and approximately 2378 cells per cell line (Jurkat scramble, VASP-KO or normal Jurkat cells) were evaluated.

### Flow cytometry

#### Quantitation of p8-transfer

The transfer of p8 was quantified as described earlier [29]. Briefly, Jurkat T-cells were pre-stained with CMAC (20 μM in serum free medium) for 45 min at 37°C and washed three times in medium. At 48 h after transfection of 1x10^7^ Jurkat T-cells with p8-HA expression plasmids or pME (control), 1x10^6^ transfected cells as well as 1x10^6^ pre-stained Jurkat T-cells (Jurkat-CMAC) were either washed in PBS, fixed in 2% PFA (20 min, 20°C) individually and mixed afterwards (time point 0 h), or cells were co-cultured for 1 h or 24 h at 37°C prior to fixation. For intracellular staining, fixed cells were washed in PBS with 0.5% FCS and 2 mM EDTA, permeabilized by addition of 0.5% saponin (Sigma Aldrich/Merck) and stained with anti-HA-APC or the respective isotype-matched control antibody mouse IgG1-APC (both Miltenyi Biotec; 1:40 in permeabilization buffer) for 10 min at 20°C. After two washing steps with 0.3% saponin, cells were resuspended in buffer without saponin and analyzed with the BD LSRII flow cytometer. First, living cells were gated based on forward scatter (FSC) versus side scatter (SSC). Second, cells were discriminated between CMAC-negative p8 donor cells and CMAC-positive acceptor Jurkat T-cells. Third, HA-specific fluorescence was plotted against the SSC either in CMAC-negative donor cells (efficiency of transfection) or in CMAC-positive acceptor cells (absolute transfer of p8). The relative p8 transfer was calculated as described earlier [29].

#### Measuring of p8 surface expression

1x10^7^ stably transduced Jurkat T-cells (Jurkat scramble or VASP-KO, *see* Knockout of VASP by CRISPR/Cas9) were transfected with Tax expression plasmids pEF-1α-Tax, p8-HA, VASP-FLAG (50 μg each) or both p8-HA and VASP-FLAG. Cells transfected with empty vectors pME and pEF-1α served as control. All samples were replenished with the respective empty vectors to 100 μg. At 48 h post transfection, cells were washed in PBS with 0.5% FCS and 2 mM EDTA (wash buffer) and stained without permeabilization in wash buffer using anti-HA-APC or the respective isotype-matched control antibody mouse IgG1-APC (both Miltenyi Biotec, 1:40, 10 min, 20°C). After another two washing steps, cells were analyzed with the BD LSRII flow cytometer.

#### Cell-cell aggregation assay

Flow cytometry-based cell-cell aggregation assays were performed as described previously [52]. Briefly, 1x10^7^ stably transduced Jurkat T-cells (Jurkat scramble or VASP-KO, *see* Knockout of VASP by CRISPR/Cas9) were transfected with Tax expression plasmids pEF-1α-Tax, p8-HA, VASP-FLAG (50 μg each) or both p8-HA and VASP-FLAG. Cells transfected with empty vectors pME and pEF-1α served as control. All samples were replenished with the respective empty vectors to 100 μg. To measure cell-cell aggregation, 1x10^6^ transfected Jurkat T-cells were co-cultured with 1x10^6^ Raji/CD4^+^ B-cells at 48 h post transfection for 1 h at 37°C. After washing steps with PBS and fixation with 2% PFA (15 min, 25°C), unspecific binding was blocked by washing once with PBS/ 5% FCS. Cells were stained with anti-CD3-AlexaFluor700 (BioLegend, San Diego, CA, USA) in PBS/ 5% FCS to stain Jurkat T-cells and with anti-HLA-DR-PacificBlue (BioLegend) in PBS/ 5% FCS to stain Raji/CD4^+^ B-cells (10 min, 4°C). The percentage of double-stained cells (CD3^+^/HLADR^+^), representing cell-aggregates of Jurkat T-cells with Raji/CD4^+^ B-cells, was determined using a BD LSRII flow cytometer (BD Biosciences, San Jose, CA, USA) and values were normalized on the total number of CD3^+^ cells (Jurkat T-cells).

#### Quantitation of Gag p19 transfer

1x10^6^ MT-2 cells (shNonsense, shVASP2, shVASP3) were co-cultured with 1x10^6^ Jurkat T-cells for 1 h at 37°C. Cells were stained using mouse monoclonal antibodies anti-Gag p19 (ZeptoMetrix Corporation, Buffalo, NY, USA), anti-mouse AlexaFluor 647-conjugated secondary antibodies (LifeTechnologies), and anti-CD25-PE (Miltenyi Biotech) as described earlier [52]. Cells were discriminated by their different size (FSC/SSC) and by CD25-staining (Jurkat: CD25-negative; MT-2: CD25-positive). The percentage of Gag-positive cells within Jurkat T-cells was examined to measure Gag transfer from MT-2 to Jurkat T-cells.

### Knockout of VASP by CRISPR/Cas9

Two different guide-RNA sequences targeting *VASP* were designed with the CRISPR gRNA Design tool (DNA2.0, Atum, Newark, CA, USA) and were: GCCAATTCCTTTCGCGTCGT (VASP1) or GGAAGAGATGAACGCCATGC (VASP2). Oligonucleotides were inserted into the lentiCRISPRv2 vector [53] (kind gift from Feng Zhang (Addgene plasmid #52961)) replacing the 2 kb filler sequence by *BsmB*I restriction enzyme, resulting in the vectors lentiCRISPRv2-VASP-guide1 (VASP1) and lentiCRISPRv2-VASP-guide2 (VASP2). The control vector lentiCRISPRv2-scramble-guide (scramble) carrying a non-specific RNA sequence was described earlier [19]. For lentiviral production, 5x10^6^ 293T cells were seeded in 10 cm dishes and 24 h later, cells were transfected with GeneJuice transfection reagent according to the manufacturer’s protocol using a total amount of 15 μg DNA: 6 μg of CRISPR vector (either 6 μg scramble, or 3 μg VASP1 + 3 μg VASP2), 6 μg psPAX2 and 3 μg pMD2.G. At 72 h post transfection, lentivirus-containing supernatants were filtered (0.45 μm) and concentrated (4000 g, 15 min) using *Amicon Ultra-15 Centrifugal Filter Concentrator Ultracell-100K* (Merck Millipore, Darmstadt, Germany). 1x10^6^ Jurkat T-cells were spin-infected with 1.5 ml concentrated virus (150 g, 1.5 h, 32°C). Cells were selected starting at 48 h after transduction by addition of 0.5 μg/ml puromycin to the cell culture medium. At 14 and 28 d post transduction, knockout of VASP was verified by immunoblot and Sanger sequencing and cell stocks were generated. Vitality of stable VASP-KO Jurkat cells was screened by propidium iodide (PI) stains. Briefly, dead cells were displayed by PI staining (10 μM). Jurkat cells treated with the topoisomerase II inhibitor etoposide (15 μM, 24 h) dissolved in DMSO served as positive control.

### small hairpin (sh) RNA mediated knockdown of VASP

Retroviral shRNA expression vectors pSiren-RetroQ-IRES-EGFP-shVASP1 (shVASP1), shVASP2, and shVASP3 were constructed. Oligonucleotides for shRNAs were designed with the RNAi Target Sequence Selector (Clontech). They contained (5' to 3') a *BamH*I site, the respective siRNA sequence (bold), a loop region, the complementary siRNA sequence (bold), an RNA polymerase III termination sequence, an *Mlu*I restriction enzyme site (italicized), and an *EcoR*I cloning site (shVASP1-fwd: 5′-gatcc**GAGCCAAACTCAGGAAAGT**ttcaagaga**ACTTTCCTGAGTTTGGCTC**tttttt*ACGCGT*g-3′; shVASP1-rev: 5′- aattc*ACGCGT*aaaaaa**GAGCCAAACTC AGGAAAGT**tctcttgaa**ACTTTCCTGAGTTTGGCTC**g-3′; shVASP2-fwd: 5'-gatcc**GCAGTGATTACTCGGACCTA**ttcaagaga**TAGGTCCGAGTAATCACTG**tttttt*ACGCGT*g-3'; shVASP2rev: 5'-aattc*ACGCGT*aaaaaa**CAGTGATTACTCGGACCTA** tctcttgaa**TAGGTCCGAGTAATCACTGC**g-3'; shVASP3-fwd: 5'-gatcc**GGATGAAGTCGTCTTCTTCT**tcaagaga**GAAGAAGACGACTTCATCC**tttttt*ACGCGT*g-3'; shVASP3rev: 5'-aattc*ACGCGT*aaaaaa**GGATGAAGTCGTCTTCTTC** tctcttgaa**GAAGAAGACGACTTCATCC**g-3'). Oligonucleotides were annealed in H_2_O by heating to 95°C for 30 s followed by stepwise cooling to room temperature. Double-stranded oligonucleotides were thereafter inserted into the retroviral vector pSiren-RetroQ-IRES-EGFP-shNonsense (shNonsense) [75] using T4 ligase (DNA Ligation kit, TaKaRa Biomedicals, Gennevilliers, France) after removal of the shNonsense fragment via *BamH*I and *EcoR*I restriction sites. The resulting shRNA expression plasmids shVASP1, shVASP2 and shVASP3 target the *VASP* coding sequence (gene bank accession number NM_003370.3) at positions 694, 1013, and 955, respectively. shRNA vectors were used to transiently transfect Jurkat T-cells (shNonsense, shVASP1) or to transduce HTLV-1-infected MT-2 cells (shNonsense, shVASP2, shVASP3). To produce retroviral particles, 5x10^6^ GP-293 cells (Clontech, Mountain View, CA, USA) were seeded in 10 cm dishes. 24 h later, cells were transfected with 10 μg of the retroviral expression plasmids shNonsense, shVASP2, or shVASP3 and 5 μg of pMD2.G encoding vesicular stomatitis virus glycoprotein (VSV-G) using *Gene Juice* reagent. At 72 h post transfection, supernatants containing viral particles were filtered (0.45 μm) and enriched using *Amicon Ultra-15 Centrifugal Filter Concentrator Ultracell-100K* (4000g, 15min). Viral particles in ca. 1 ml of culture medium were used to infect 3x10^6^ HTLV-1-infected MT-2 cells by spin infection (1500 rpm, 1.5 h, 32°C). Afterwards, cells were kept at a density of 500.000 cells per ml. From day 3 after transduction, MT-2 cells were selected with culture medium containing 0.5 μg/ml puromycin (Life Technologies) and monitored by flow cytometry and western blot. After 24 d, selected cells were frozen at -80°C to generate cell stocks.

### Gag p19 ELISA

MT-2 cells were seeded at 5x10^5^ cells/ml. After 48 h, supernatants of MT-2 cells were sterile filtrated by passing through a 0.45 μm filter. HTLV-1 release was determined using Gag p19 ELISA according to the manufacturer’s instruction (ZeptoMetrix Corporation). MT-2 cells treated with cytochalasin D (5 μM; 48 h) dissolved in DMSO were used as control. Values were obtained using Softmax Pro Version 5.3 software (MDS Analytical Technologies, Sunnyvale, CA, USA). At least three independent experiments, each performed in duplicate, were performed.

### Statistics

Microsoft Excel was used for statistical analysis using the t-test. P<0.05 was considered to be significant.

## Supporting information

S1 Fig Stains of FLAG-VASP (blue) and p8-HA (red), the merge of both stains and transmitted light are shown. ROIs are shown and highlighted in insets. Solid arrows indicate co-localizations of p8-HA and FLAG-VASP. Open arrows highlight a protrusive structure. Graphs show the fluorescence intensities of FLAG-VASP- and p8-HA-specific fluorescence along the ROI.
(TIF)

S2 Fig p8 is transferred to target T-cells via VASP-containing protrusions. **(A-B)** Jurkat T-cells were co-transfected with expression plasmids p8-HA, FLAG-VASP and pMACS-LNGFR. After 48 h, transfected cells were enriched by magnetic separation using LNGFR-specific microbeads and co-cultured with untransfected Jurkat T-cells pre-stained with the live cell marker Calcein (green) on poly-L-lysine-coated coverslips for 30 min at 37°C. Immunofluorescence stainings of FLAG-VASP (blue), p8-HA (red) and the merge of all stainings are shown. Additionally, a merge showing an overlay with transmitted light is depicted. White arrow: p8 co-localizing with VASP in a protrusion; black arrow: p8 in co-cultured target Jurkat T-cell.
(TIF)

S3 Fig Repression of endogenous and overexpressed VASP reduces transfer of p8 to target T-cells. Jurkat T-cells were transfected with expression plasmids p8-HA and pMACS-LNGFR. Additionally, FLAG-VASP or pEF (mock), shRNAs targeting VASP, or control shRNAs (shNonsense) were co-transfected. After 48 h, transfected cells were enriched by magnetic separation using anti-LNGFR-specific microbeads. Purified Jurkat T-cells were co-cultivated with acceptor Jurkat T-cells pre-stained with CellTracker Blue Dye CMAC on poly-L-lysine coated glass slides for 1 h at 37°C. Thereafter, cells were stained with HA- and FLAG-specific antibodies and the respective secondary antibodies. Slides were covered with *ProLong Gold antifade reagent* and analyzed by confocal microscopy. The numbers of cells expressing p8 (red) within the acceptor Jurkat T-cells (blue) were counted (see white circle in blow up as example) and are displayed in Fig 6C.
(TIF)

S4 Fig Validation of stable Jurkat VASP-KO cell lines. **(A)** Immunoblot analysis of Jurkat T-cells at days 14 and 28 post transduction with the CRISPR/Cas9 vectors scramble (Jurkat scramble) and VASP1+VASP2 (Jurkat VASP-KO). **(B)** Propidiumiodide (PI; 10 μM) staining of the indicated cell lines. Jurkat cells treated with the topoisomerase II inhibitor etoposide (15 μM, 24 h) dissolved in DMSO served as positive control. The percentage of living cells (PI-negative) is indicated and values were compared using Student’s t-test (**, p<0.01).
(TIF)

S5 Fig Validation of VASP and p8 protein expression. Immunoblot analysis was performed using protein lysates obtained from **(A)** Jurkat scramble, VASP-KO and normal Jurkat cells transfected as indicated and used for assays quantitating cell-cell protrusions (Fig 8B and 8C) or **(B)** from transfected Jurkat-scramble and VASP-KO cells used for cell-cell-aggregation assays (Fig 8D). Representative blots are shown.
(TIF)
