# Supplementary figures and images for: Transfer of HTLV-1 p8 and Gag to target T-cells depends on VASP, a novel interaction partner of p8
Source: PLoS Pathog. 2020 Sep 30;16(9):e1008879. doi: 10.1371/journal.ppat.1008879 (PMC7526893; doi:10.1371/journal.ppat.1008879)

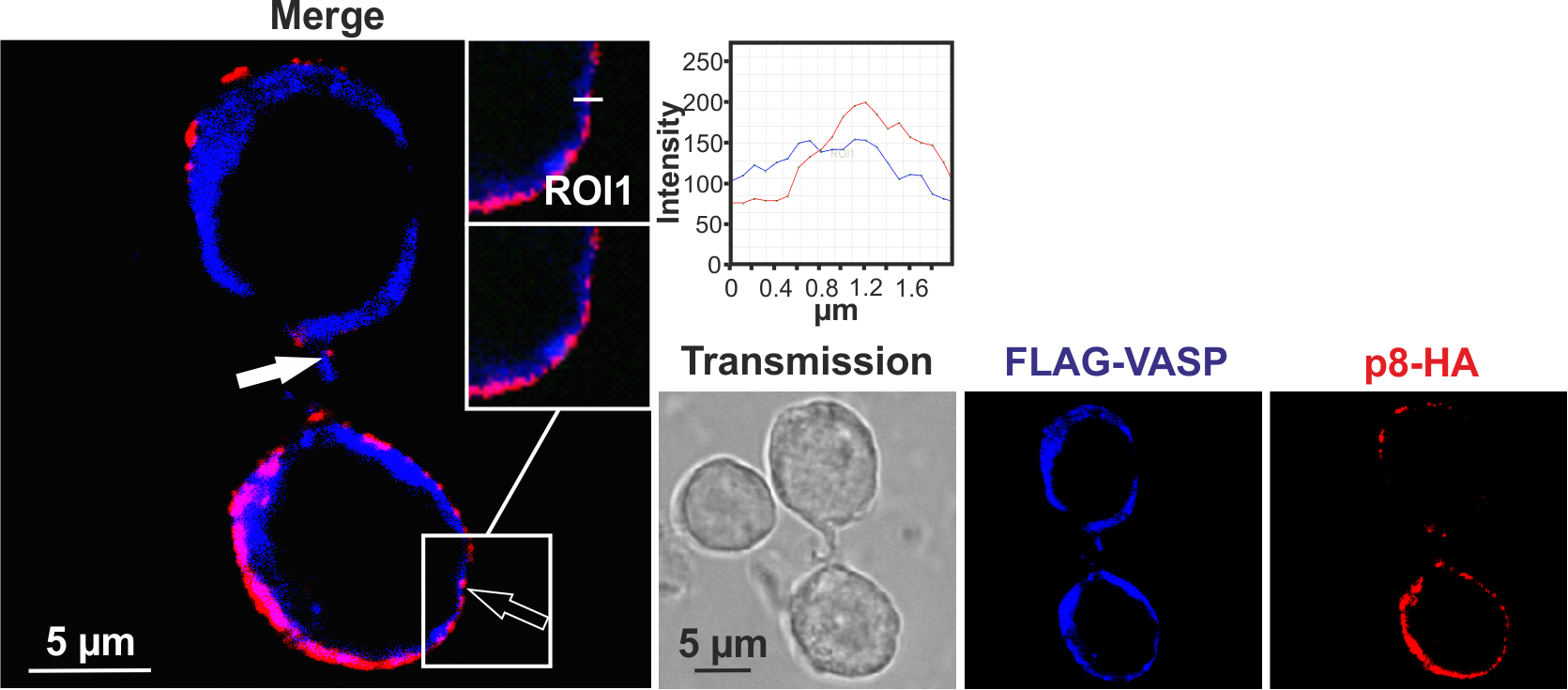

Supplement: S1 Fig — Stains of FLAG-VASP (blue) and p8-HA (red), the merge of both stains and transmitted light are shown. ROIs are shown and highlighted in insets. Solid arrows indicate co-localizations of p8-HA and FLAG-VASP. Open arrows highlight a protrusive structure. Graphs show the fluorescence intensities of FLAG-VASP- and p8-HA-specific fluorescence along the ROI. (TIF) [file ppat.1008879.s001.tif]

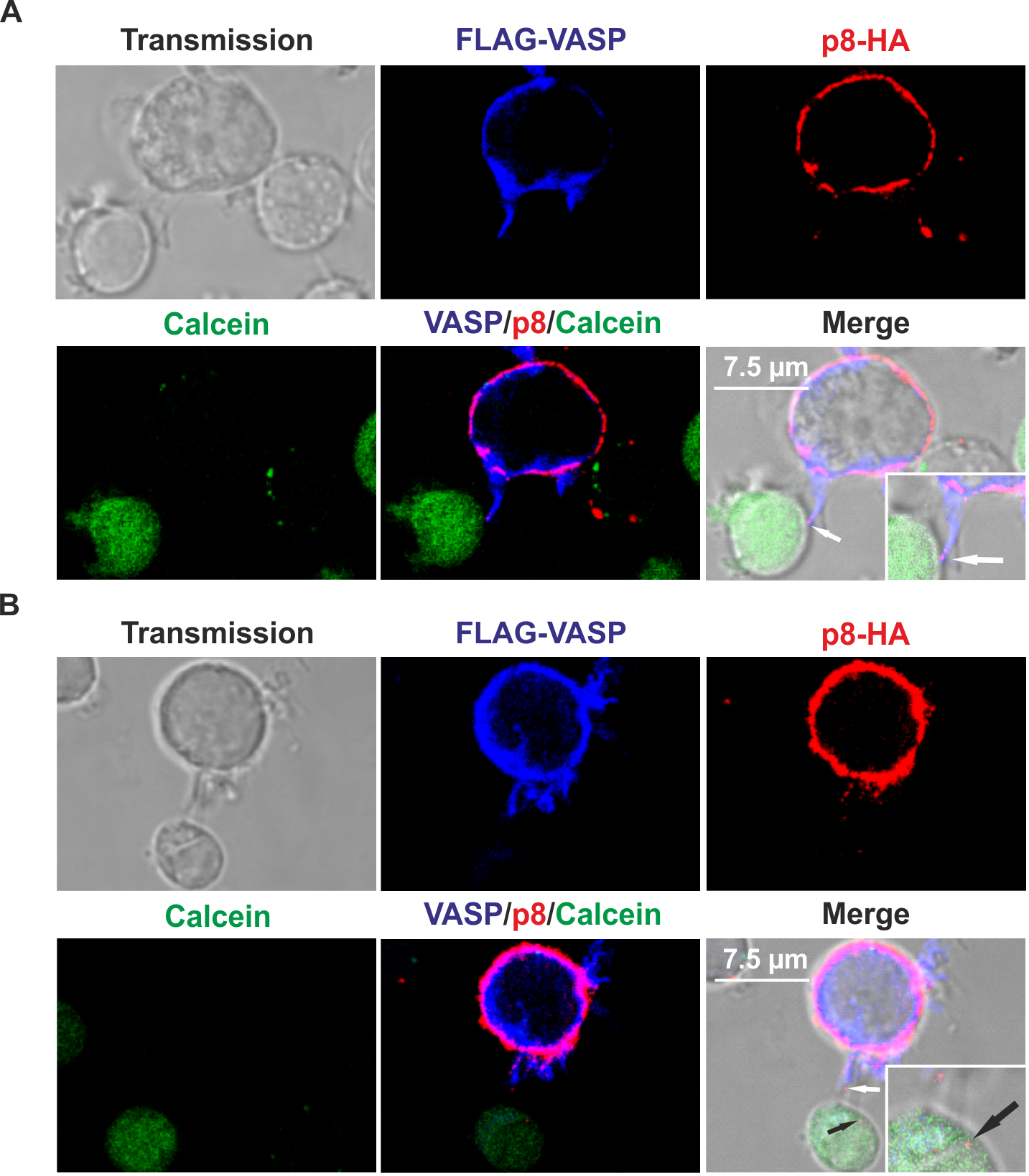

Supplement: S2 Fig — (A-B) Jurkat T-cells were co-transfected with expression plasmids p8-HA, FLAG-VASP and pMACS-LNGFR. After 48 h, transfected cells were enriched by magnetic separation using LNGFR-specific microbeads and co-cultured with untransfected Jurkat T-cells pre-stained with the live cell marker Calcein (green) on poly-L-lysine-coated coverslips for 30 min at 37°C. Immunofluorescence stainings of FLAG-VASP (blue), p8-HA (red) and the merge of all stainings are shown. Additionally, a merge showing an overlay with transmitted light is depicted. White arrow: p8 co-localizing with VASP in a protrusion; black arrow: p8 in co-cultured target Jurkat T-cell. (TIF) [file ppat.1008879.s002.tif]

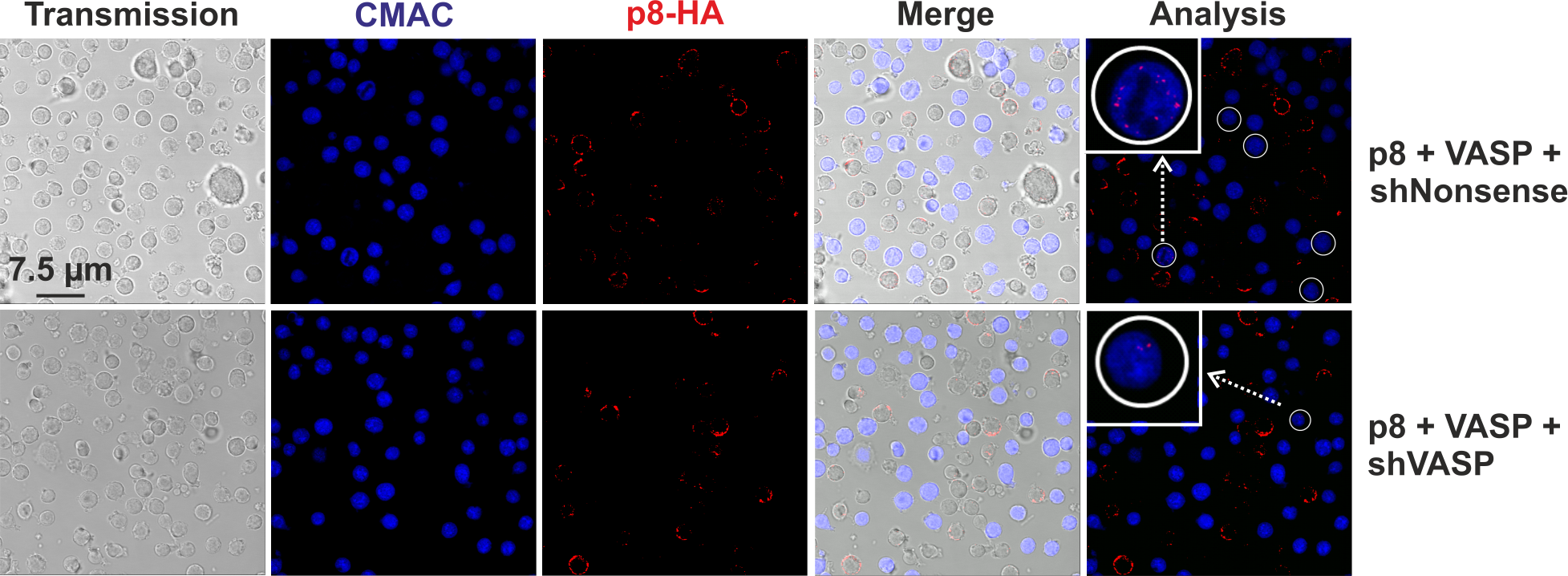

Supplement: S3 Fig — Jurkat T-cells were transfected with expression plasmids p8-HA and pMACS-LNGFR. Additionally, FLAG-VASP or pEF (mock), shRNAs targeting VASP, or control shRNAs (shNonsense) were co-transfected. After 48 h, transfected cells were enriched by magnetic separation using anti-LNGFR-specific microbeads. Purified Jurkat T-cells were co-cultivated with acceptor Jurkat T-cells pre-stained with CellTracker Blue Dye CMAC on poly-L-lysine coated glass slides for 1 h at 37°C. Thereafter, cells were stained with HA- and FLAG-specific antibodies and the respective secondary antibodies. Slides were covered with ProLong Gold antifade reagent and analyzed by confocal microscopy. The numbers of cells expressing p8 (red) within the acceptor Jurkat T-cells (blue) were counted (see white circle in blow up as example) and are displayed in Fig 6C. (TIF) [file ppat.1008879.s003.tif]

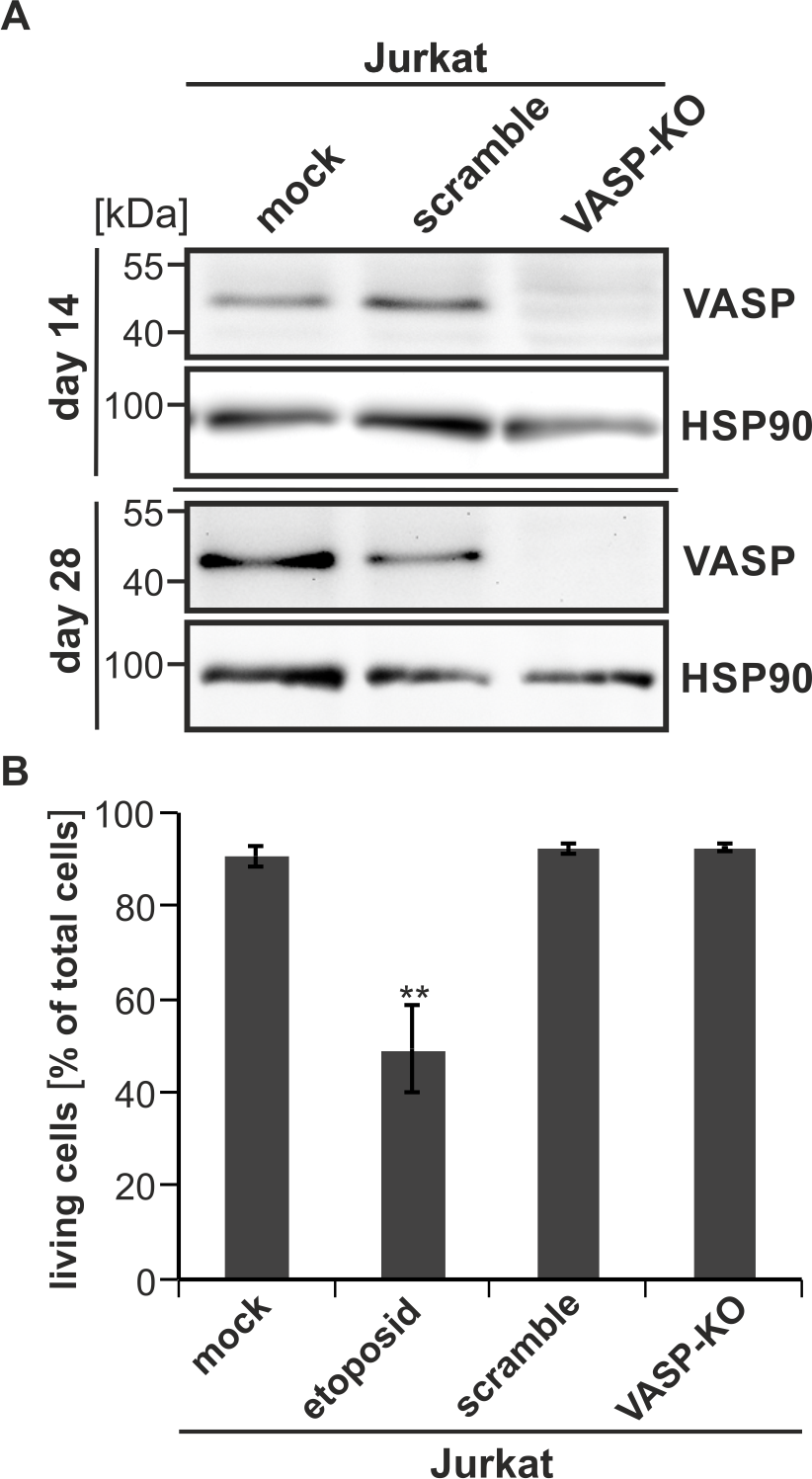

Supplement: S4 Fig — (A) Immunoblot analysis of Jurkat T-cells at days 14 and 28 post transduction with the CRISPR/Cas9 vectors scramble (Jurkat scramble) and VASP1+VASP2 (Jurkat VASP-KO). (B) Propidiumiodide (PI; 10 μM) staining of the indicated cell lines. Jurkat cells treated with the topoisomerase II inhibitor etoposide (15 μM, 24 h) dissolved in DMSO served as positive control. The percentage of living cells (PI-negative) is indicated and values were compared using Student’s t-test (**, p<0.01). (TIF) [file ppat.1008879.s004.tif]

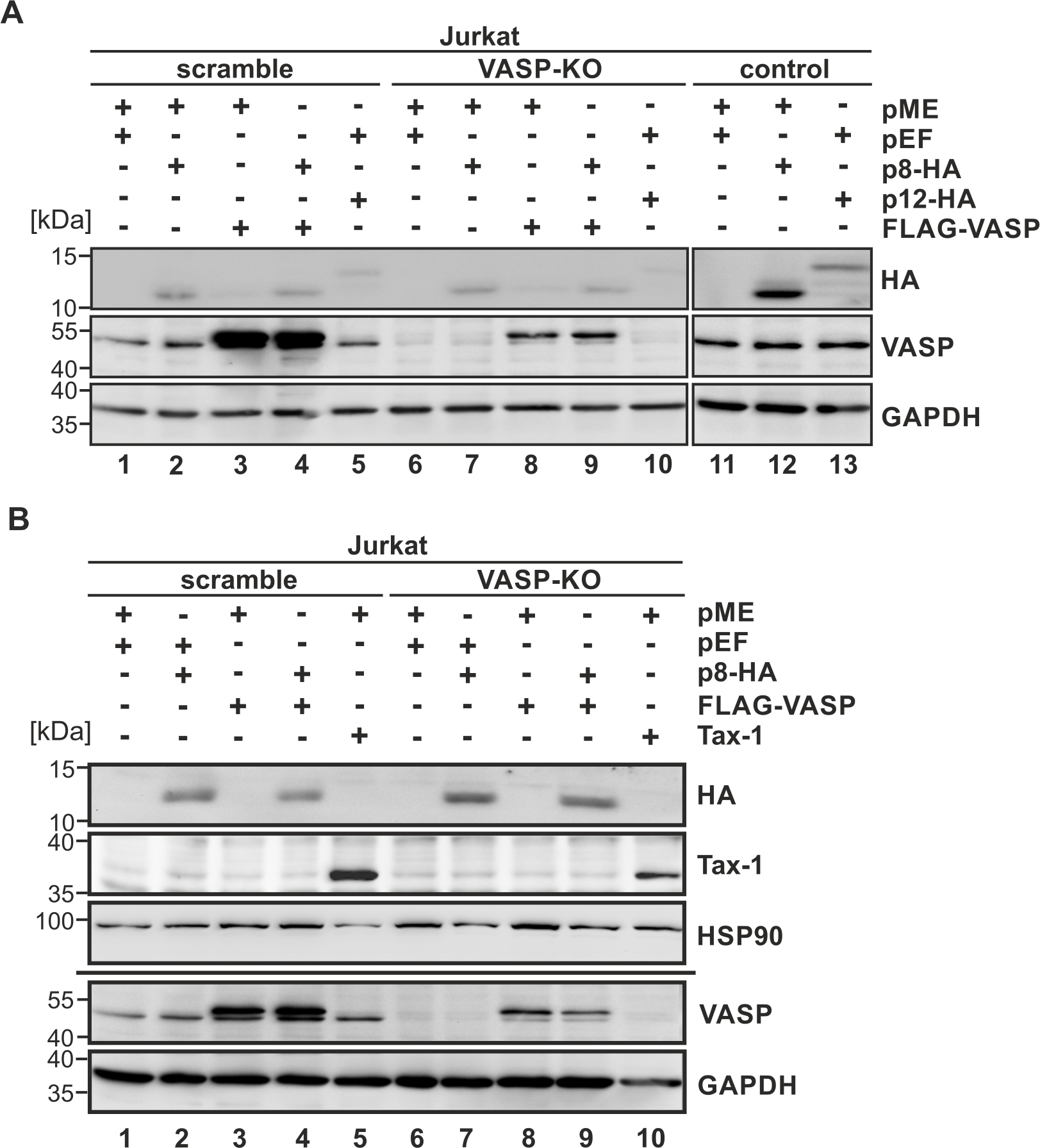

Supplement: S5 Fig — Immunoblot analysis was performed using protein lysates obtained from (A) Jurkat scramble, VASP-KO and normal Jurkat cells transfected as indicated and used for assays quantitating cell-cell protrusions (Fig 8B and 8C) or (B) from transfected Jurkat-scramble and VASP-KO cells used for cell-cell-aggregation assays (Fig 8D). Representative blots are shown. (TIF) [file ppat.1008879.s005.tif]
